# Post-translational modifications in the Protein Data Bank

**DOI:** 10.1107/S2059798324007794

**Published:** 2024-08-29

**Authors:** Lucy C. Schofield, Jordan S. Dialpuri, Garib N. Murshudov, Jon Agirre

**Affiliations:** a York Structural Biology Laboratory, Department of Chemistry University of York York United Kingdom; b MRC Laboratory of Molecular Biology University of Cambridge Cambridge United Kingdom; Linköping University, Sweden

**Keywords:** post-translational modifications, Protein Data Bank, glycosylation, phosphorylation, acetylation

## Abstract

Proteins frequently undergo covalent modification at the post-translational level, which involves the covalent attachment of chemical groups onto amino acids. This can entail the singular or multiple addition of small groups, such as phosphorylation; long-chain modifications, such as glycosylation; small proteins, such as ubiquitination; as well as the interconversion of chemical groups, such as the formation of pyroglutamic acid. These post-translational modifications (PTMs) are essential for the normal functioning of cells, as they can alter the physicochemical properties of amino acids and therefore influence enzymatic activity, protein localization, protein–protein interactions and protein stability. Despite their inherent importance, accurately depicting PTMs in experimental studies of protein structures often poses a challenge. This review highlights the role of PTMs in protein structures, as well as the prevalence of PTMs in the Protein Data Bank, directing the reader to accurately built examples suitable for use as a modelling reference.

## Abstract 2

This review explores the importance of post-translational modifications (PTMs) in the Protein Data Bank and identifies examples of well modelled PTMs.

## 1. Introduction

Protein post-translational modifications (PTMs) are covalent modifications of amino acids that can alter the physicochemical properties, and therefore the function, of a protein. As a result, PTMs are essential for regulating various biochemical processes in cells, such as protein localization, epigenetic regulation and cell signalling (Smotrys & Linder, 2004 ▸; Wang *et al.*, 2019 ▸; Rocks *et al.*, 2005 ▸). Dysregulated PTMs have been shown to have a role in many human diseases, including hyperglycosylation in cancer (Thomas *et al.*, 2021 ▸), elevated histone acetylation in diabetes (Wang *et al.*, 2019 ▸) and hyperphosphorylation in neurodegenerative diseases (Basheer *et al.*, 2023 ▸). Understanding PTMs, particularly in three-dimensional protein structures, is essential for furthering the knowledge of general protein function, the molecular basis of disease, and drug discovery (Bhullar *et al.*, 2018 ▸; Dekker *et al.*, 2014 ▸; Copeland, 2018 ▸).

PTMs can encompass the addition of small molecules, such as phosphorylation and methylation; long-chain modifications, such as glycosylation and lipidation; small proteins, such as in ubiquitination and SUMOylation; as well as the interconversion of chemical groups, such as the formation of isopeptide bonds, pyroglutamic acid and citrulline. The most common targets of post-translational modification are the side chains of amino acid residues, as well as the N- and C-termini of the protein chain. These modifications can alter the chemistry of target amino acids, including size, charge, surface area and hydrophobicity, which can lead to changes in protein properties, such as conformation, protein–protein interactions and enzyme activity (Betts *et al.*, 2017 ▸; van den Bedem & Wilson, 2019 ▸; T *et al.*, 2018 ▸). These modifications are often reversible and dynamic, with a tendency to occur in disordered regions that are often surface-accessible, allowing amino acids to dynamically fit into the catalytic site of modifying enzymes (Pang *et al.*, 2007 ▸; Xie *et al.*, 2007 ▸). Many PTMs do not exist in isolation (Venne *et al.*, 2014 ▸), as proteins can undergo multiple modifications at various sites that can influence the actions of each other, known as PTM crosstalk (Leutert *et al.*, 2021 ▸).

Traditionally, PTMs have been detected and characterized using experimental methods, such as western blotting, and mass spectrometry (Wilkins *et al.*, 1999 ▸). As a result of these techniques, the volume of PTM experimental data necessitated the creation of open, easy-access databases. The dbPTM is a database that compiles information on PTMs, which includes both experimentally determined PTM sites as well as putative sites (Li *et al.*, 2022 ▸). The experimentally determined sites are derived from existing PTM databases and extracted from research articles; these are mapped to UniProt entries to ensure a nonredundant data set (UniProt Consortium, 2021 ▸). Putative sites are derived from the UniProt Knowledgebase (UniProtKB), which predicts these sites based on sequence similarity or evolutionary potential (UniProt Consortium, 2021 ▸; Li *et al.*, 2022 ▸). The total number of PTM sites in the dbPTM therefore includes a nonredundant list of sites that have been identified either experimentally or putatively (Li *et al.*, 2022 ▸). The dbPTM highlights phosphorylation, ubiquitination, acetylation and glycosylation as some of the most common PTM sites (shown in Table 1 ▸). The large collection of PTM data in these databases paved the way for PTM prediction tools, such as *NetPhos*, *DeepAcet*, *HydLoc* and *DeepKhib* (Blom *et al.*, 1999 ▸; Wu *et al.*, 2019 ▸; Huang, Chen *et al.*, 2020 ▸; Zhang *et al.*, 2020 ▸). These are commonly based on machine-learning approaches trained on experimental data that predict PTM sites based on sequence features, structural properties and evolutionary conservation (Kumar *et al.*, 2017 ▸; Wu *et al.*, 2019 ▸; Huang, Chen *et al.*, 2020 ▸; Blom *et al.*, 1999 ▸; Bludau *et al.*, 2022 ▸). In parallel, the availability of highly accurate whole-proteome structure predictions has made it possible to map putative phosphorylation, ubiquitination and acetylation PTM sites onto 3D models, allowing algorithms to refine predictions based on factors such as solvent-accessibility (Bludau *et al.*, 2022 ▸; Joosten & Agirre, 2022 ▸).

Whilst this allows the identification of experimentally determined and putative sites, it does not provide detailed structural information. As PTMs contain information relevant to protein function that is not encoded in the protein sequence, the structure of these modifications is crucial to understand protein structure and function. Macromolecular X-ray crystallography (MX), cryo-electron microscopy (cryo-EM) single-particle analysis (SPA) and nuclear magnetic resonance (NMR) are powerful tools for determining protein structures (Kumar *et al.*, 2020 ▸; Atanasova *et al.*, 2020 ▸; Reid *et al.*, 2004 ▸). However, they face limitations when studying modified proteins, as many post-translational modifications are labile, and the dynamic nature of PTMs leads to non-uniform modification sites, contributing to structural heterogeneity. For a given molecule, each site is either modified or not, and consequently the averaging over all of the unit cells of a crystal in X-ray crystallography, or particles in cryo-EM, can result in a lower apparent occupancy of PTMs, as some molecules may be modified while others are not. PTMs also typically occur in highly flexible solvent-exposed regions and frequently introduce conformational variability into proteins, which can lead to increased disorder at the protein surface (Deller *et al.*, 2016 ▸). In fact, protein glycosylation modifications are frequently removed from glycoproteins before performing structural studies in order to avoid these complexities (Agirre, 2017 ▸; Deller *et al.*, 2016 ▸; Atanasova *et al.*, 2020 ▸). While the presence of PTMs may introduce challenges in structure determination, it is essential to consider the functional significance of these modifications, as PTMs play pivotal roles in modulating protein conformation, stability and dynamics, thereby influencing protein function and regulation. As a result, incorporating PTMs into structural studies offers a more comprehensive view into protein structure, dynamics and biological relevance, enhancing the understanding of protein function.

## 2. Biochemistry of PTMs

When considering the modelling of PTMs within a protein structure, certain biochemical aspects should be considered. Understanding which amino acids can undergo modifications is vital, as most PTMs occur on specific amino acid residues. PTMs can also occur at consensus sequences or motifs, which denote specific amino acid patterns within a protein that act as recognition sites for the enzymes responsible for the modification. These motifs are often essential for the modification to take place, as is seen in *N*-glycosylation, where the consensus motif is N-*X*-S/T (where *X* represents any amino acid other than proline). These can be strict motifs, as for *N*-glycosylation, or weaker motifs, as for phosphorylation (Bludau *et al.*, 2022 ▸). Moreover, the wider structural context of the modification site is essential, such as its location within hydrophobic or disordered regions (Pang *et al.*, 2007 ▸).

Here, key biochemical information for the most relevant small-molecule PTMs and long-chain PTMs will be explored; only examples where electron density can be calculated from deposited diffraction data and a model will be showcased, along with the Chemical Component Dictionary (CCD) identifiers used by the Protein Data Bank (PDB) to represent them (Westbrook *et al.*, 2015 ▸; Berman *et al.*, 2000 ▸). For each model, omit maps were calculated to validate the presence of the modification in the electron density. Omit maps were calculated using MTZ files downloaded from the RCSB PDB (Berman *et al.*, 2000 ▸) by removing the modification followed by re-refinement with *REFMAC* using randomization, which was used to shake the remaining model (Murshudov *et al.*, 2011 ▸). The calculation of omit maps was used to reduce model bias by ensuring that the observed electron density was not influenced by prior assumptions about the presence of the modification or structure. By excluding the region of interest, the resulting omit map provides a more accurate indication of the PTM. All 3D figures were produced using *CCP*4*mg* (McNicholas *et al.*, 2011 ▸), which is part of the *CCP*4 software suite (Agirre *et al.*, 2023 ▸).

### 2.1. Phosphorylation

**Figure 1:**
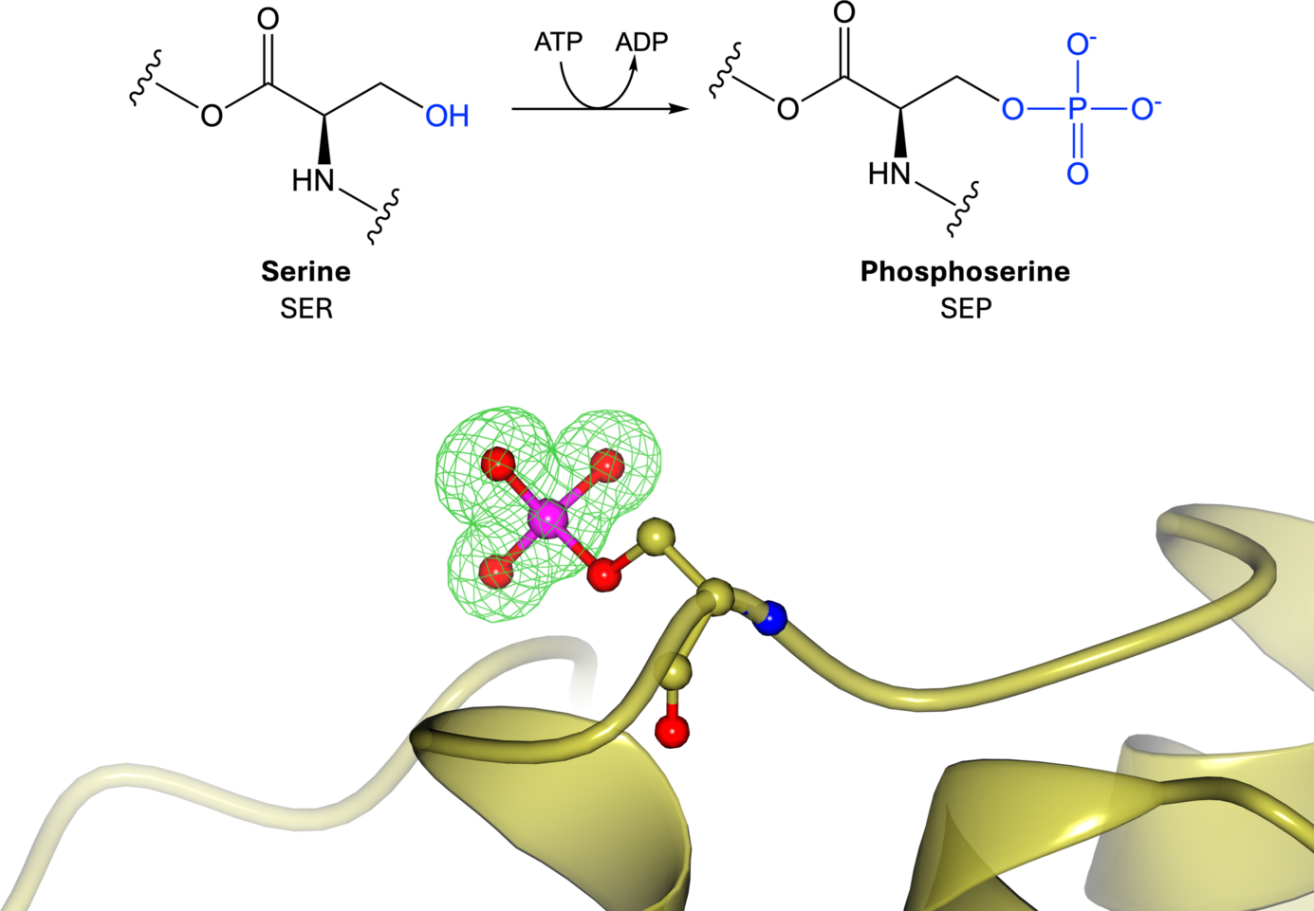
Phosphorylation. Top: phosphorylation of serine involves the addition of a phosphate group donated by ATP to the side-chain hydroxyl group. Bottom: phosphoserine (PDB entry 5n3h; Sadowsky *et al.*, 2011 ▸; CCD code SEP). Positive omit density is shown in green at 3σ for the modified residue. The rest of the protein chain is represented by a yellow ribbon model.

Protein phosphorylation is one of the most abundant and well studied PTMs in the proteome. It involves the reversible addition of a phosphate group from a nucleoside triphosphate, typically ATP, to a polar amino acid side chain via kinase enzymes (Fig. 1 ▸). Phosphorylation most commonly occurs on serine (Fig. 1 ▸), threonine or tyrosine residues (Supplementary Fig. S1), but can occur on many other amino acids (Li *et al.*, 2022 ▸). There is no strict consensus motif for phosphorylation, although individual kinases do recognize specific motifs (Miller & Turk, 2016 ▸). Phosphorylation sites often occur in disordered regions, at protein–protein interaction faces and within loop and hinge regions, affecting protein conformation, protein–protein interactions and protein stability (Betts *et al.*, 2017 ▸; T *et al.*, 2018 ▸; Qin *et al.*, 2021 ▸; Durek *et al.*, 2009 ▸; Rieloff & Skepö, 2020 ▸). The addition of a phosphate group introduces a large, dianionic group that offers a new site to form hydrogen bonds or salt bridges, which can alter protein interactions or create new binding sites (Johnson & Lewis, 2001 ▸). Phosphorylation is important for the function of many proteins, including the activation of enzymes, transcription factors and protein receptors (Betts *et al.*, 2017 ▸; T *et al.*, 2018 ▸; Mayr & Montminy, 2001 ▸). It is therefore implicated in several human diseases, including cancer, in which the tyrosine kinase family encompass the largest number of oncoproteins (Singh *et al.*, 2017 ▸), as well as in Alzheimer’s disease, where the altered protein phosphorylation states of several proteins, including the amyloid-β protein precursor and tau protein, are closely associated with protein aggregation (Kumar *et al.*, 2011 ▸; Despres *et al.*, 2017 ▸).

### 2.2. Methylation

**Figure 2:**
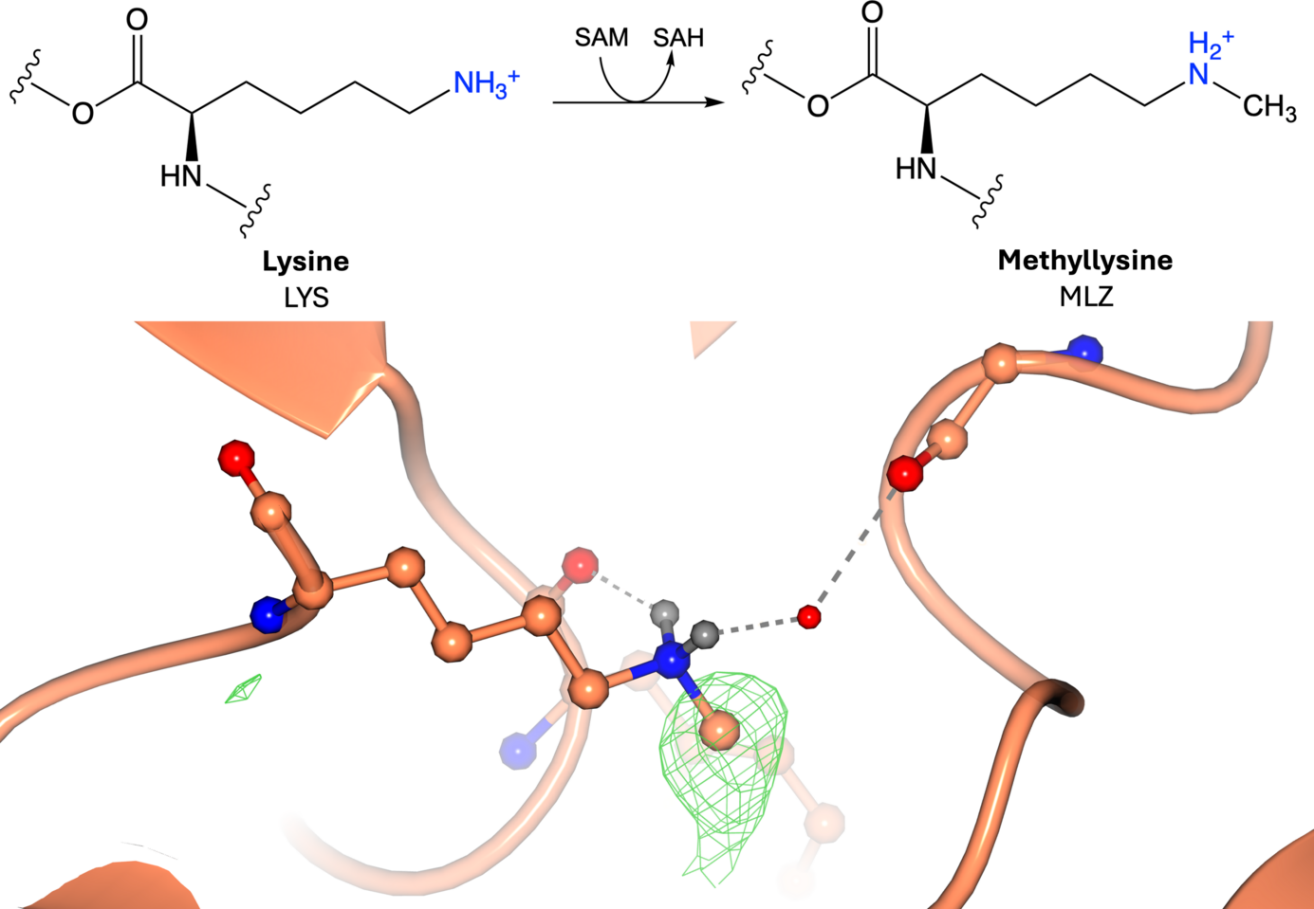
Methylation. Top: methylation of lysine involves the addition of a methyl group donated by *S*-adenosylmethionine (SAM) to the side-chain amino group. Bottom: methyllysine (PDB entry 3kmt; Wei & Zhou, 2010 ▸; CCD code MLZ). Positive omit density is shown in green at 3σ for the modified residue. The rest of the protein chain is represented by an orange ribbon model. The environment surrounding the modification is shown, indicating that the NZ atom is protonated (hydrogenation of the NZ atom was performed using *Coot*; Emsley *et al.*, 2010 ▸). Hydrogen bonds are displayed as grey dashed lines.

Protein methylation involves the reversible addition of a methyl group to the amino group of an amino acid, often donated by *S*-adenosyl-l-methionine via methyltransferase enzymes (Fig. 2 ▸; Małecki *et al.*, 2022 ▸). Lysine (Fig. 2 ▸) and arginine (Supplementary Fig. S2) are the most frequent targets of protein methylation, but it can also occur on other amino acids (Li *et al.*, 2022 ▸). The ɛ-amino group of lysine can accept up to three methyl groups, yielding mono-, di- or trimethylated states (Małecki *et al.*, 2022 ▸). The guanidino group of arginine can be methylated on one or on both N atoms, yielding monomethylarginine or dimethylarginine (asymmetric or symmetric) (Małecki *et al.*, 2022 ▸). Lysine methylation does not have a well defined consensus sequence, whereas arginine methylation commonly occurs in glycine-rich and arginine-rich regions known as GAR motifs (Wooderchak *et al.*, 2008 ▸; Lorton & Shechter, 2019 ▸; Daily *et al.*, 2005 ▸). The N-terminal methylation of amino acids can also occur by the action of N-terminal methyltransferases, with substrates often containing the consensus motif *X*-P-K/R (*X* = S/P/A/G) after the removal of the initiator methionine (Diaz *et al.*, 2021 ▸). Generally, methylation occurs in disordered protein regions, but it can also be found in ordered regions (Narasumani & Harrison, 2018 ▸). Methylation increases the bulkiness and alters the hydrogen-bonding capacity of the modified residues, which can affect protein stability, subcellular localization, binding affinity and protein–protein interactions (Liu *et al.*, 2023 ▸). Both lysine and arginine methylation are particularly abundant in the N-terminal, flexible tails of histone proteins, and result in an epigenetic mark that controls gene expression and chromatin state (Bannister & Kouzarides, 2011 ▸). Arginine methylation can also target several nonhistone proteins that regulate processes such as DNA repair and RNA splicing (Wei *et al.*, 2021 ▸; Brobbey *et al.*, 2022 ▸). Protein methylation therefore has a regulatory role in many cellular processes, including gene transcription and DNA repair, and can contribute to neurological disorders, cancer and ageing (Liu *et al.*, 2023 ▸).

### 2.3. Hydroxylation

**Figure 3:**
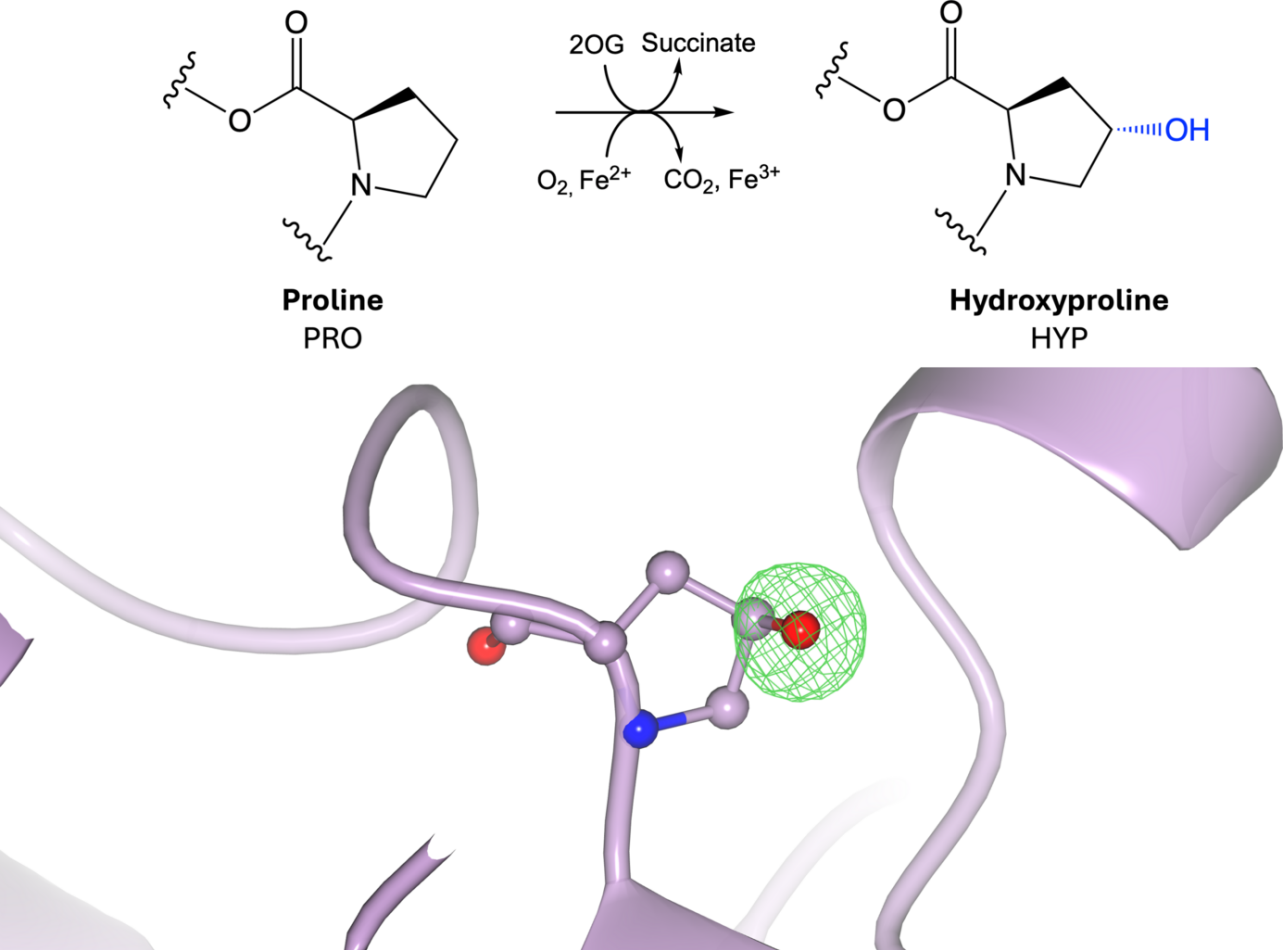
Hydroxylation. Top: hydroxylation of proline involves the addition of a hydroxyl group donated by 2-oxoglutarate (2OG) to the side-chain pyrrolidine ring. Bottom: hydroxyproline (PDB entry 1gk8; Taylor *et al.*, 2001 ▸; CCD code HYP). Positive omit density is shown in green at 3σ for the modified residue. The rest of the protein chain is represented by a purple ribbon model.

Hydroxylation is an oxidation reaction in which a carbon–hydrogen bond is oxidized into a carbon–hydroxyl bond via hydroxylase enzymes (Fig. 3 ▸). Proline is the most frequently hydroxylated residue (Fig. 3 ▸), followed by lysine (Supplementary Fig. S3); however, other residues can also undergo hydroxylation (Li *et al.*, 2022 ▸). Proline hydroxylation can occur either on the γ-carbon, forming 3-hydroxyproline, or on the β-carbon, forming 4-hydroxyproline, whilst lysine hydroxylation occurs on the δ-carbon, forming 5-hydroxy­lysine (Tak *et al.*, 2019 ▸). There is no known consensus motif for hydroxylation sites, although they tend to occur in surface-accessible, intrinsically disordered regions of proteins (Ismail *et al.*, 2016 ▸). Protein hydroxylation increases the hydrophilicity of the amino acids, allowing hydroxylated residues to become more water-soluble, which can impact protein structure and function (Varma *et al.*, 2021 ▸). Both hydroxy­proline and hydroxylysine are present in collagen, where they play important roles in water solubility and the formation of triple-helical structures found in collagen fibrils, and act as precursors for subsequent PTMs such as glycosylation (Varma *et al.*, 2021 ▸; Stawikowski *et al.*, 2014 ▸). Hydroxylation also plays a role in hypoxia signalling by regulating hypoxia-inducible factor, where prolyl hydroxylation marks the protein for ubiquitination and subsequent degradation (Bruick & McKnight, 2001 ▸); this has been shown to play a role in tumour suppression or promotion (Strocchi *et al.*, 2022 ▸).

### 2.4. Acetylation

**Figure 4:**
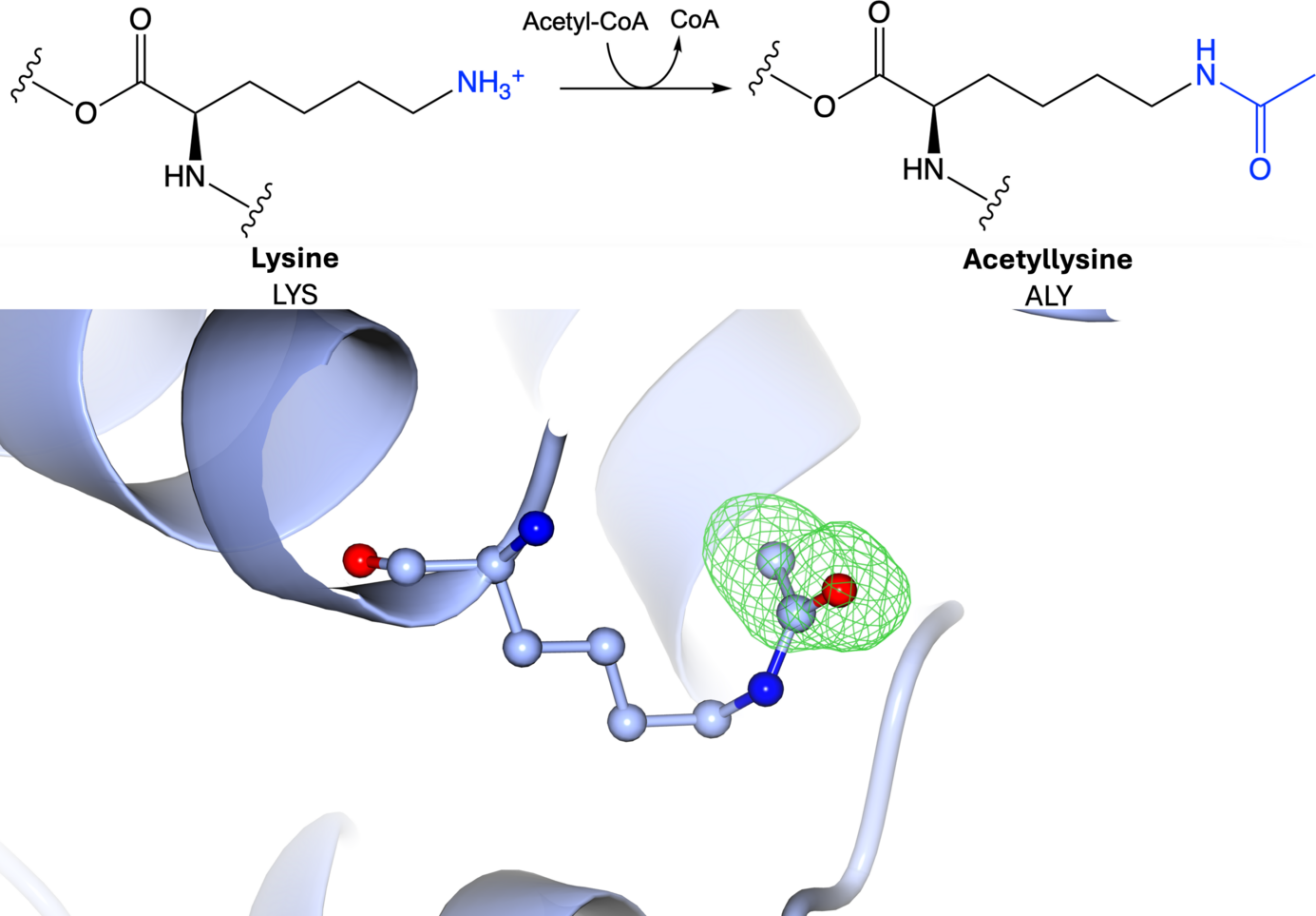
Acetylation. Top: acetylation of lysine involves the addition of an acetyl group donated by acetyl-CoA to the side-chain amino group. Bottom: acetyllysine (PDB entry 5e2f; Y. Kim, G. Joachimiak, M. Endres, G. Babnigg & A. Joachimiak, unpublished work; CCD code ALY). Positive omit density is shown in green at 3σ for the modified residue. The rest of the protein chain is represented by a blue ribbon model.

Protein acetylation involves the reversible addition of an acetyl group onto an amino acid (Fig. 4 ▸). Acetylation most frequently occurs on lysine (Fig. 4 ▸) and alanine (Supplementary Fig. S4), as well as methionine and serine, but other amino acids can also be acetylated (Li *et al.*, 2022 ▸). Lysine acetylation occurs on the ɛ-amino group of lysine side chains, which is important for gene transcription through histone acetylation (Davie, 1998 ▸). Histone acetyltransferases catalyse the addition of acetyl groups onto histone lysine residues via acetyl-CoA, while histone deacetylases remove them, meaning the modification is reversible (Davie, 1998 ▸). Protein acetylation can also occur irreversibly on the free α-amino group at the protein N-terminus via N-terminal acetyltransferases (Varland *et al.*, 2015 ▸). A global consensus motif for acetylation has not been identified, although lysine-acetylation motifs have been identified in specific organisms (Weinert *et al.*, 2011 ▸, 2013 ▸; Choudhary *et al.*, 2009 ▸; Okanishi *et al.*, 2013 ▸; Lundby *et al.*, 2012 ▸; Crosby & Escalante-Semerena, 2014 ▸; Crosby *et al.*, 2012 ▸). Acetylation neutralizes charges on amino acids, affecting the electrostatic properties of proteins. For example, the acetylation of lysine residues on histone proteins weakens the histone–DNA binding affinity, leading to chromatin relaxation, increased accessibility to DNA-binding proteins and subsequent gene transcription (Davie, 1998 ▸). As a result, lysine acetylation controls the regulation of proteins involved in many diseases, including cancer and inflammatory conditions (Bai *et al.*, 2016 ▸; Hu *et al.*, 2022 ▸). Meanwhile, N-terminal acetylation transforms a charged protein N-terminus into a hydrophobic segment, which can impact protein folding, stability, protein–protein interactions and localization (Hwang *et al.*, 2010 ▸; Trexler & Rhoades, 2012 ▸; Scott *et al.*, 2011 ▸; Behnia *et al.*, 2004 ▸). In fact, dysregulation of N-terminal acetylation has been shown to have implications in cancer, as well as in developmental disorders, including brain and heart development (Varland, Silva *et al.*, 2023 ▸; Varland, Brønstad *et al.*, 2023 ▸; Koufaris & Kirmizis, 2020 ▸).

### 2.5. Oxidation

**Figure 5:**
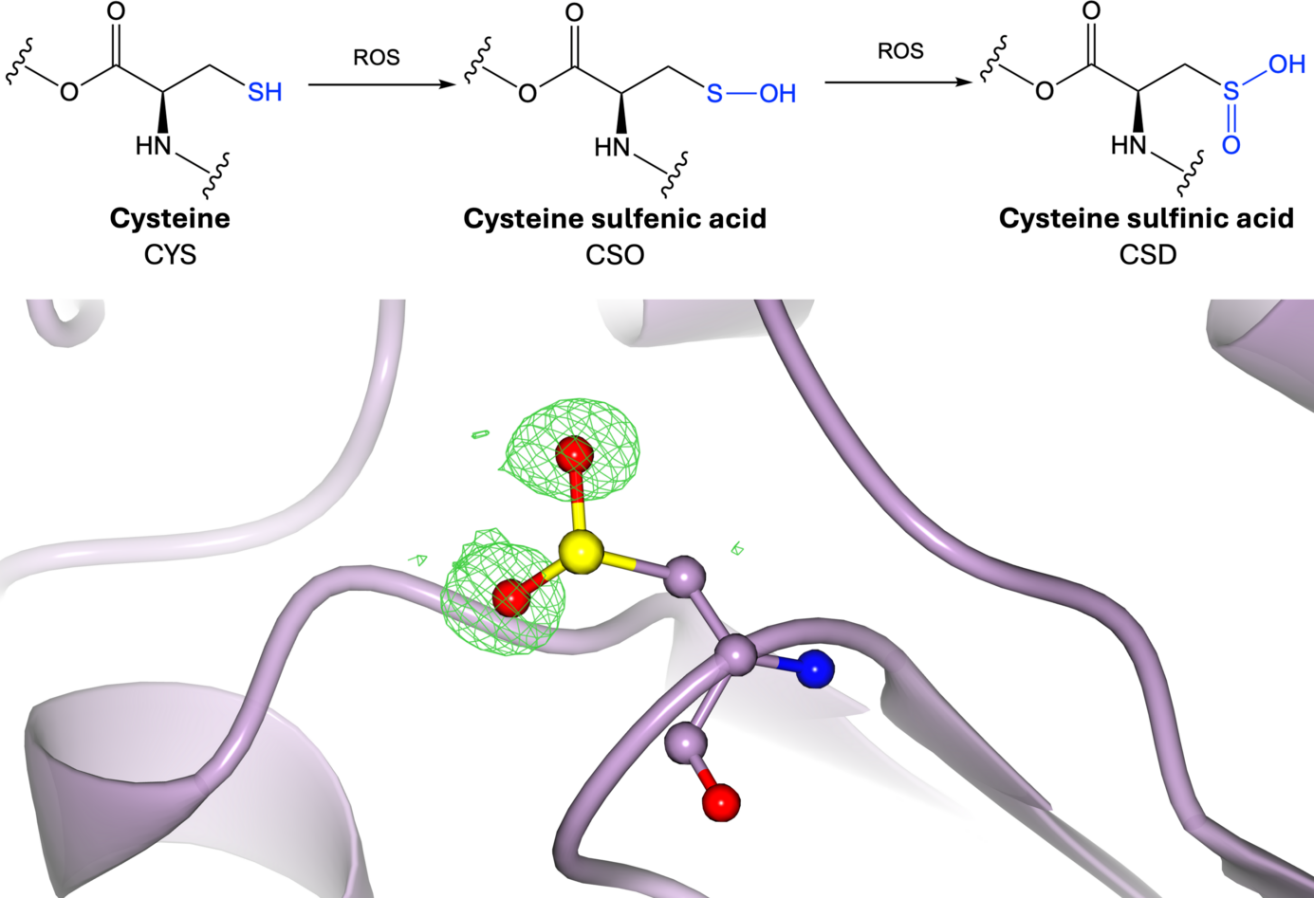
Oxidation. Top: oxidation of cysteine involves the reaction between the side-chain thiol group of cysteine and reactive oxygen species (ROS) to form cysteine sulfenic, then cysteine sulfinic acid and cysteine sulfonic acid (Supplementary Fig. S5). Bottom: cysteine sulfinic acid (PDB entry 1soa; Canet-Avilés *et al.*, 2004 ▸; CCD code CSD). Positive omit density is shown in green at 3σ for the modified residue. The rest of the protein chain is represented by a purple ribbon model.

Protein oxidation involves the addition of oxygen-containing groups to amino acid residues via reactive oxygen species (ROS; Fig. 5 ▸). This includes sulfoxidation reactions, in which reactive sulfur-containing residues such as cysteine and methionine are the targets of oxidative stress (Li *et al.*, 2022 ▸). One-electron oxidation of cysteine forms thiyl radicals, which react either with other thiols to form disulfide bonds or with O_2_ to generate thiyl peroxyl radicals (Wardman & von Sonntag, 1995 ▸). The two-electron oxidation of cysteine by oxidants forms unstable sulfenic acid, sulfinic acid (Fig. 5 ▸) and sulfonic acid species (Supplementary Fig. S5), which either yield oxyacids by hydrolysis reactions or disulfide bonds by reacting with other thiol groups (Claiborne *et al.*, 1999 ▸; Turell *et al.*, 2008 ▸). Methionine residues can also be oxidized to form methionine sulfoxide, which can be further oxidized to methionine sulfone (Hoshi & Heinemann, 2001 ▸). Furthermore, oxidant species such as superoxide can react with nitric oxide to form nitrosating species, which can lead to nitrosylated cysteine residues or nitrated tyrosine residues (Nedospasov *et al.*, 2000 ▸; Berlett *et al.*, 1996 ▸). Oxidation lacks a strict motif, although it often occurs in surface-accessible regions (Sanchez *et al.*, 2008 ▸; Yang *et al.*, 2017 ▸; Garrido Ruiz *et al.*, 2022 ▸). Oxidation of protein residues introduces a polar group, which can affect the structural properties of proteins, and cysteine oxidation has been shown to affect cell signalling and enzyme activity (van den Bedem & Wilson, 2019 ▸), whilst methionine oxidation has been shown to destabilize proteins and affect enzyme function, with implications in neurological disorders (Liu *et al.*, 2008 ▸; Mulinacci *et al.*, 2011 ▸; Chandran & Binninger, 2023 ▸).

### 2.6. Pyroglutamic acid

**Figure 6:**
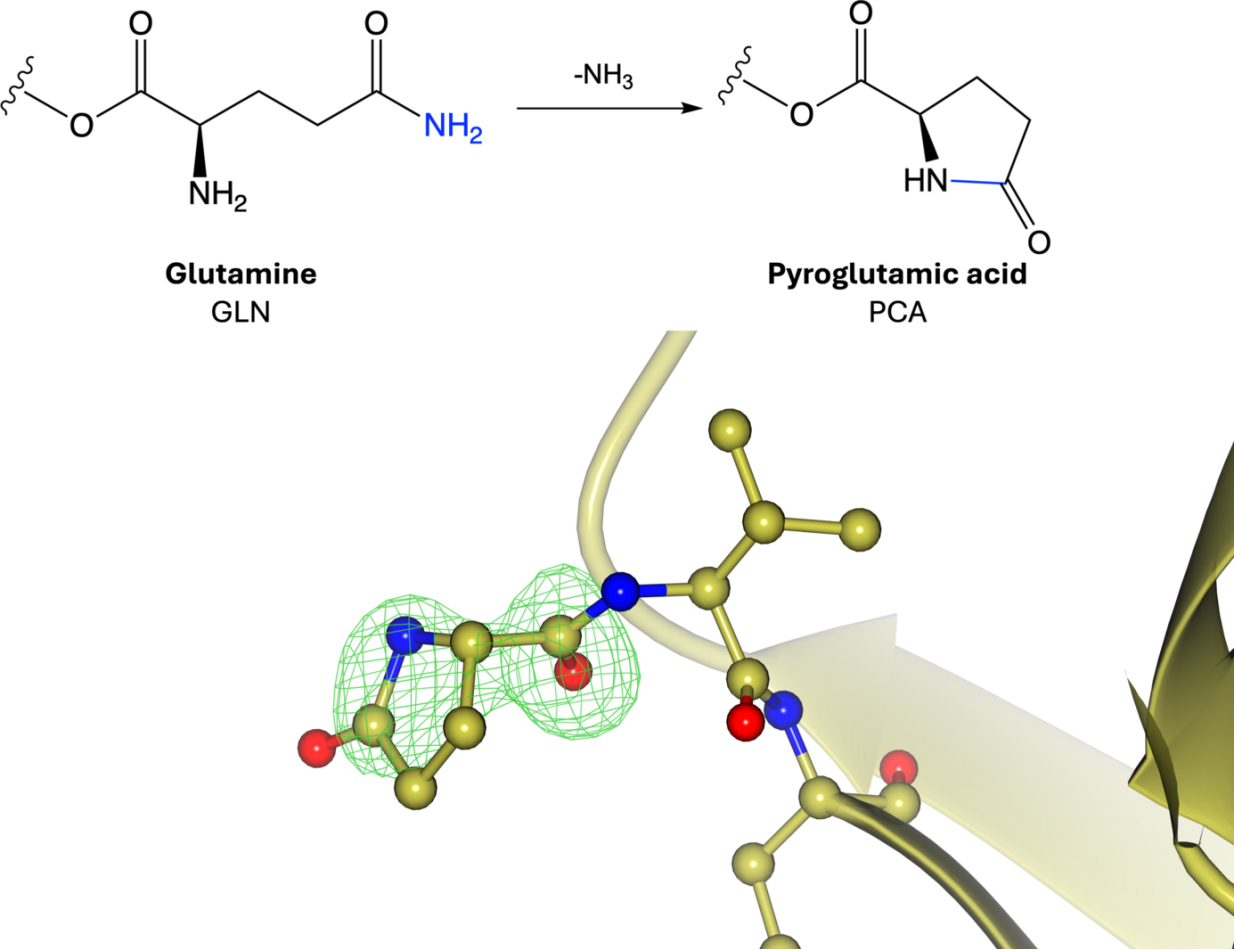
Pyroglutamic acid. Top: formation of pyroglutamic acid involves the cyclization of an N-terminal glutamine or glutamic acid. Bottom: pyroglutamic acid (PDB entry 8ojt; Davies *et al.*, 2023 ▸; CCD code PCA). The first three N-terminal residues are shown. Positive omit density is shown in green at 3σ for the modified residue. The rest of the protein chain is represented by a yellow ribbon model.

Pyroglutamic acid (also known as pyrrolidone carboxylic acid or 5-oxoproline) is an amino acid modification involving the cyclic lactam of glutamic acid or glutamine (Fig. 6 ▸, Supplementary Fig. S6; Kumar & Bachhawat, 2012 ▸; Connell & Hanes, 1956 ▸). It is formed by the cyclization of N-terminal glutamine or glutamic acid residues through enzymatic catalysis by glutaminyl cyclase, and most commonly occurs on glutamine residues (Connell & Hanes, 1956 ▸; Kumar & Bachhawat, 2012 ▸; Li *et al.*, 2022 ▸). Pyroglutamic acid can also form spontaneously under acidic conditions, which has been shown to occur in immunoglobulin structures *in vitro* (Chelius *et al.*, 2006 ▸). The lack of an identified consensus sequence for this modification can be attributed to limited research in this area, although it has been suggested that pyroglutamic acid is more likely to occur within coiled regions (Pang *et al.*, 2007 ▸). Many proteins, including antibodies, enzymes and structural proteins, have been shown to exhibit an N-terminal pyroglutamic acid (Brandt *et al.*, 1984 ▸; Chelius *et al.*, 2006 ▸). This modification has been shown to increase the half-life of antibodies and structural proteins by blocking the action of aminopeptidases, thereby reducing protein degradation (Cummins & O’Connor, 1998 ▸; Chelius *et al.*, 2006 ▸; Brandt *et al.*, 1984 ▸). In addition, it has been shown to affect protein–receptor binding and has been linked to protein aggregation in Alzheimer’s disease by increasing hydrophobicity and encouraging β-sheet formation (Gunn *et al.*, 2010 ▸; Hinkle & Tashjian, 1973 ▸).

### 2.7. Glycosylation

**Figure 7:**
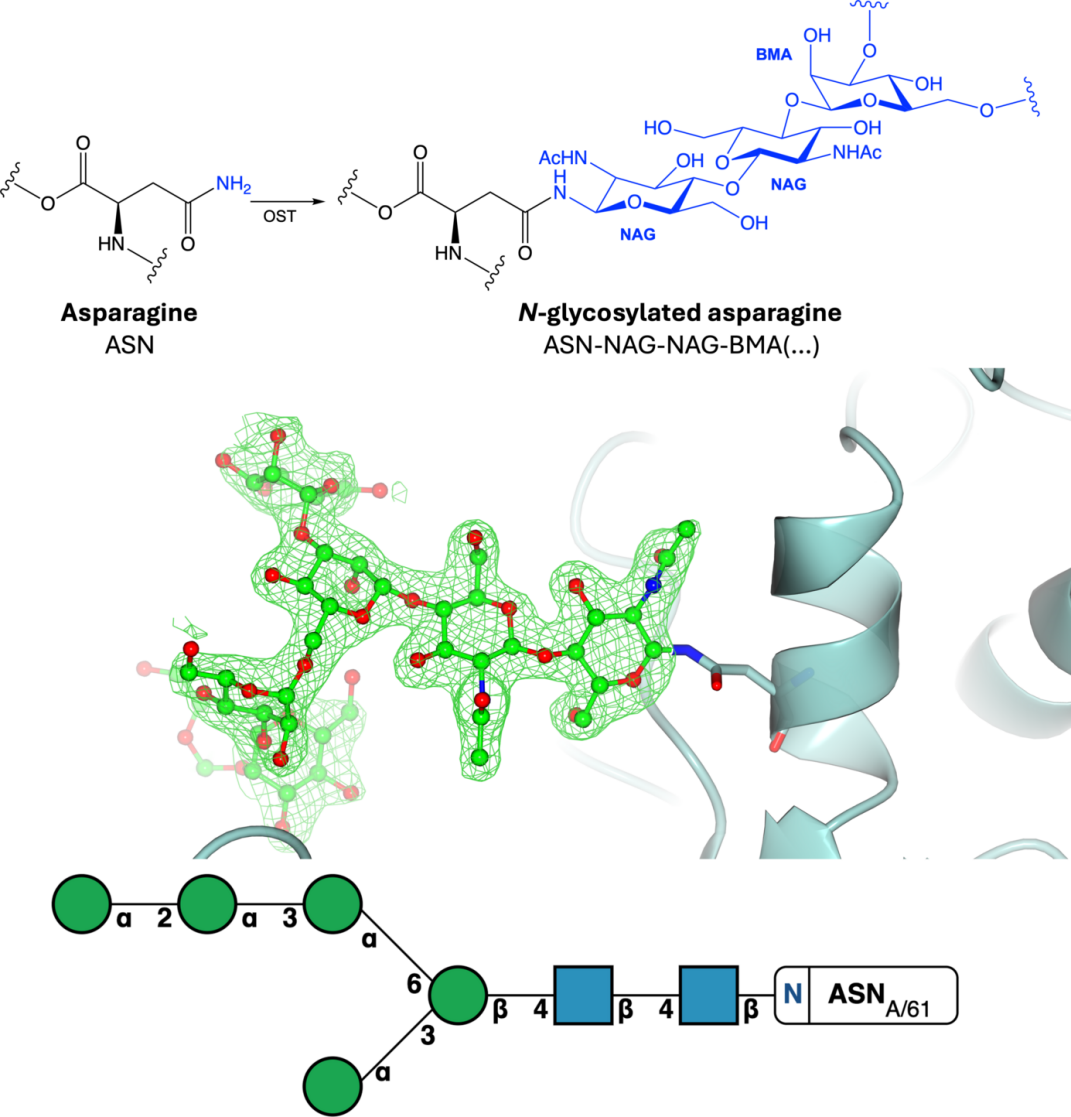
*N*-Glycosylation. Top: *N*-glycosylation of asparagine involves the addition of an *N*-glycan to the side-chain amino group via oligosaccharide transferase (OST). Middle: N-linked glycosylation (PDB entry 5fji; Agirre *et al.*, 2016 ▸). Positive omit density is shown in green at 3σ for the modified residue. The rest of the protein chain is represented by a blue ribbon model. Bottom: the symbol nomenclature for glycans (SNFG) representation is shown and was generated using the *Privateer Web App* (Dialpuri, Bagdonas, Schofield, Pham, Holland, Bond *et al.*, 2024 ▸).

**Figure 8:**
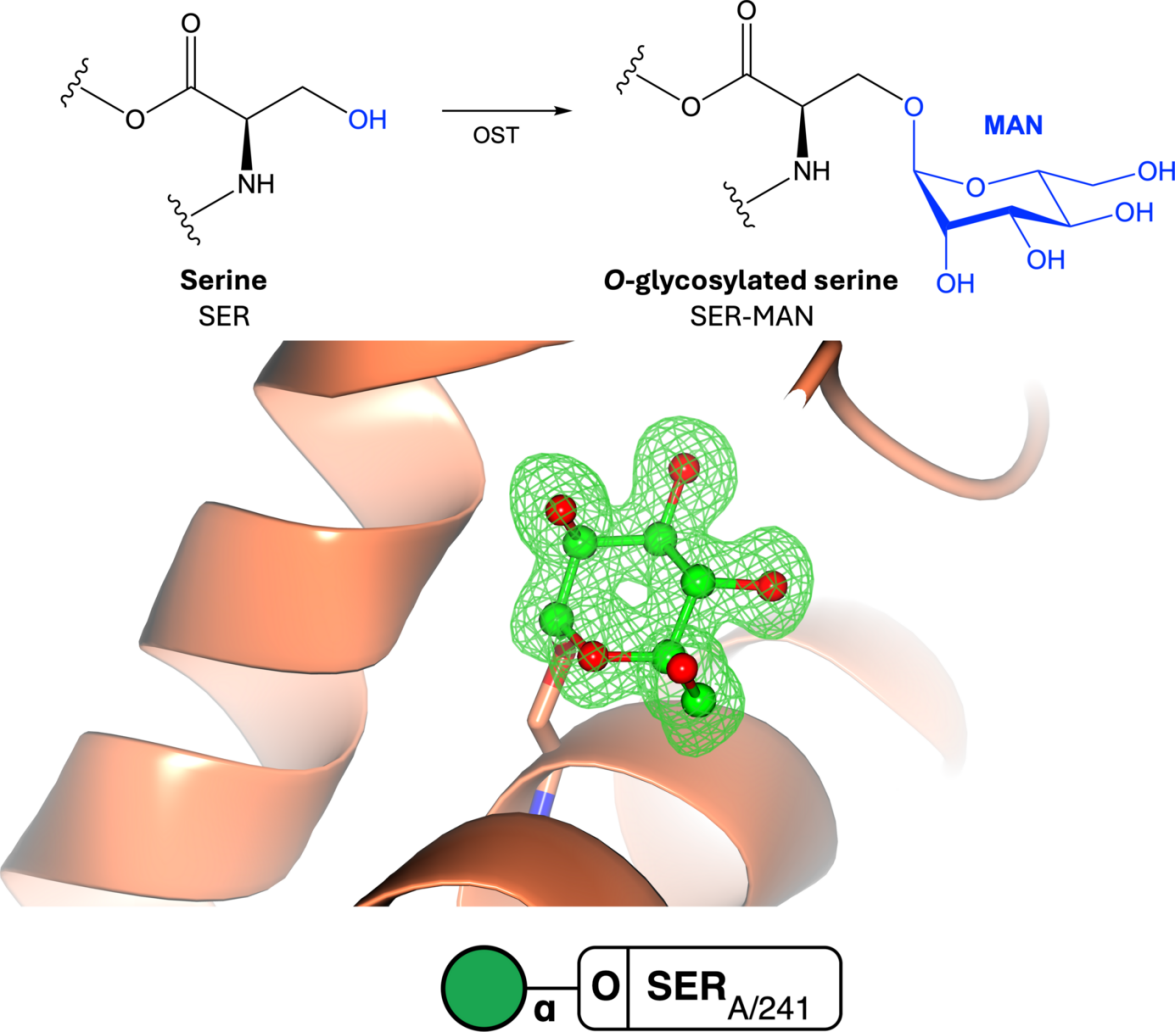
*O*-Glycosylation. Top: *O*-glycosylation of serine or threonine involves the addition of an *O*-glycan to the side-chain hydroxyl group via oligosaccharide transferase (OST). Middle: O-linked glycosylation (PDB entry 2ciw; Kühnel *et al.*, 2006 ▸). Omit density is shown in green at 3σ for the modified residue. The rest of the protein chain is represented by an orange ribbon model. Bottom: the symbol nomenclature for glycans (SNFG) representation is shown and was generated using the *Privateer Web App* (Dialpuri, Bagdonas, Schofield, Pham, Holland, Bond *et al.*, 2024 ▸).

One of the most commonly occurring and well studied PTMs is glycosylation, where an oligosaccharide moiety is covalently attached to an amino acid via a glycosidic bond, forming a glycoprotein. Protein glycosylation is often categorized into two major types: N-linked and O-linked glycosylation, with C- and S-linked glycosylation being far less frequent (Varki *et al.*, 2022 ▸). N-linked glycosylation is one of the most well studied PTMs and occurs on the side-chain N atom of an asparagine residue, with a strict consensus sequence N-*X*-S/T (where *X* is any amino acid except proline; Fig. 7 ▸, Supplementary Fig. S7), whereas *O*-glycosylation occurs on the side-chain O atom of a serine or threonine residue with no known consensus sequence (Fig. 8 ▸, Supplementary Fig. S8). Glycosylation reactions are diverse and are catalysed by numerous different enzymes that attach specific glycans to specific amino acids. Glycosylation sites are typically surface-accessible and coat the surface of many proteins, including antibodies, enzymes and cell-surface receptors (Suga *et al.*, 2018 ▸). It is estimated that at least 50% of human proteins are glycosylated (An *et al.*, 2009 ▸). Glycosylation is crucial in many biological processes, including molecular recognition and cell signalling, meaning that glycans play a critical role in human health and disease. For example, deregulation of glycosylation can contribute to several hallmarks of cancer through increased proliferation and metastasis (Purushothaman *et al.*, 2023 ▸) and play a role in viral infections such as in SARS-CoV-2, where spike glycoproteins protruding from the cell surface are necessary for viral host-cell entry (Huang, Yang *et al.*, 2020 ▸).

The study of the glycoproteome is challenging due to the multitude and diversity of glycoprotein isoforms. This is a result of complex glycan-processing events that involve subsequent trimming and modification events that occur according to the available cellular enzymes: glycoside hydrolases, glycosyl transferases and oligosaccharyl transferases. Understanding the three-dimensional structure of sugars is challenging due to their various stereochemical and anomeric conformations (Agirre, 2017 ▸; Atanasova *et al.*, 2020 ▸). Previously, the production of a correct three-dimensional structure of a glycoprotein was difficult as many refinement and validation processes relied on software written for proteins and nucleic acids, and the libraries of restraints had become outdated (Agirre, 2017 ▸; Agirre *et al.*, 2017 ▸; Atanasova *et al.*, 2020 ▸). In addition, obtaining a high-resolution structure is generally more difficult for proteins containing sugars due to glycan heterogeneity and mobility, which lead to poorer experimental data than for sugar-free structures (Agirre *et al.*, 2017 ▸; van Beusekom *et al.*, 2018 ▸).

### 2.8. Lipidation

Protein lipidation describes a series of modifications in which a lipid molecule is covalently attached to a protein. Several types of lipidation exist, including the addition of fatty acids, isoprenoids, sterols, phospholipids and glycosylphos­phatidylinositol anchors. Lipidation is crucial for cell signalling, as it modulates protein function in reaction to stimuli by increasing protein hydrophobicity. This can alter protein–membrane binding affinities, change subcellular localization, and impact protein folding, stability and protein–protein interactions (Rocks *et al.*, 2005 ▸; Tanaka *et al.*, 1995 ▸). The most extensively studied lipidation types include the addition of fatty-acid chains (palmitoylation and myristoylation) and prenylation (addition of isoprenoids, including farnesylation and geranylgeranylation). Whilst proteins are more or less ordered and structured, lipids are flexible and may have partial occupancy. In addition, lipids have complex conformations and complex torsion angles. Whilst saturated lipids are extremely flexible due to free rotation around their many single bonds, unsaturated lipids have *cis*–*trans* stereochemistry that should be considered; recently, it has been shown that many PDB structures containing lipids have incorrect *cis*–*trans* stereochemistry (Waibl *et al.*, 2022 ▸).

### 2.9. Lipidation: palmitoylation

**Figure 9:**
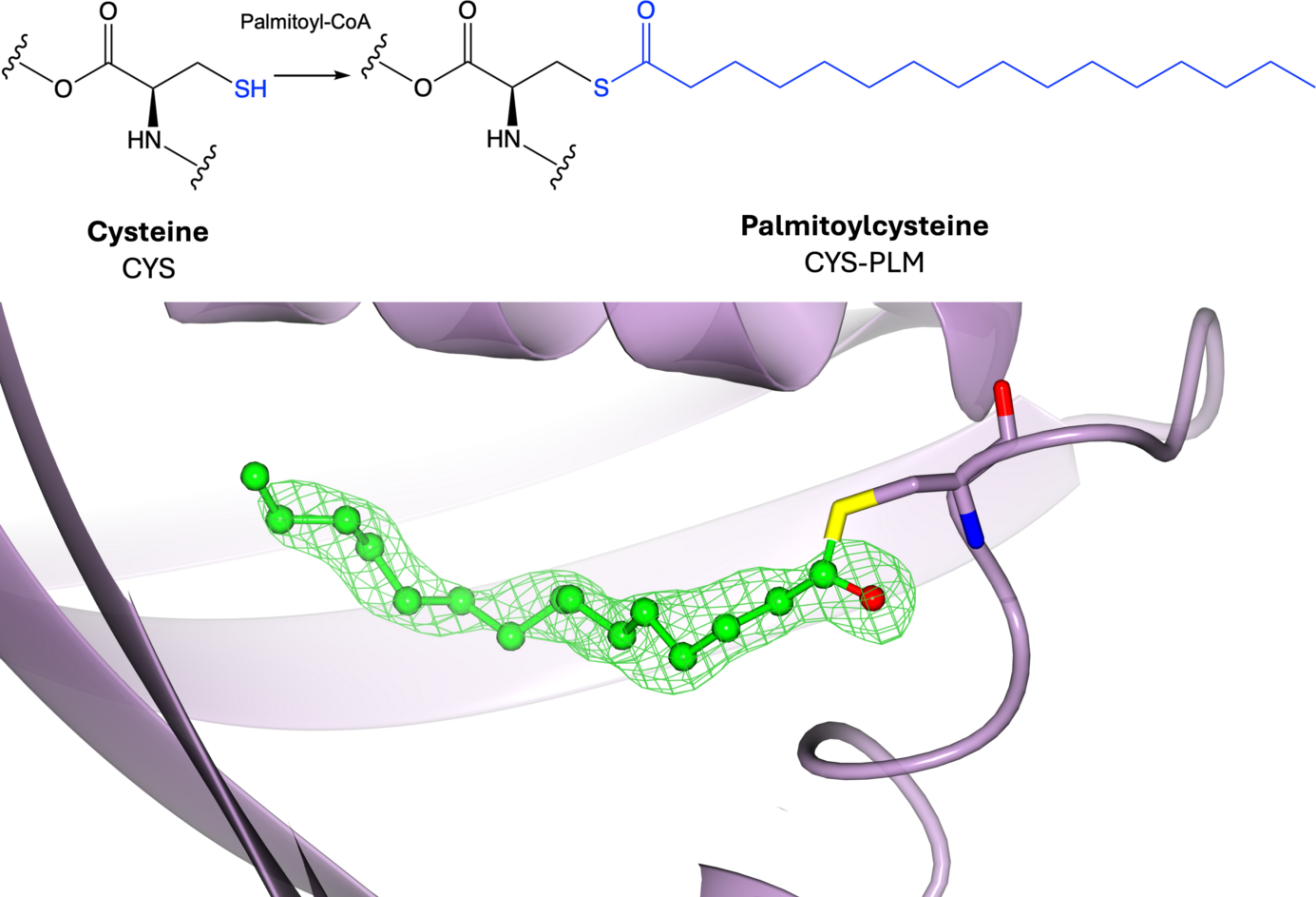
Palmitoylation. Top: palmitoylation of cysteine involves the addition of a palmitoyl group donated by palmitoyl-CoA to the side-chain thiol group. Bottom: palmitoylcysteine (PDB entry 2w3y; Quevillon-Cheruel *et al.*, 2009 ▸; CCD code PLM). Positive omit density is shown in green at 3σ for the modified residue. The rest of the protein chain is represented by a purple ribbon model.

Palmitoylation describes the covalent attachment of a 16-carbon fatty-acid palmitoyl group to a protein (Fig. 9 ▸, Supplementary Fig. S9). The most common type of palmitoylation is *S*-palmitoylation, which involves the reversible attachment of palmitate from palmitoyl-CoA to the thiol group of a cysteine residue via a thioester linkage by palmitoyl acyltransferases (Li *et al.*, 2022 ▸). Less commonly, irreversible *N*-palmitoylation can occur at the N-terminus of Hedgehog proteins (Buglino & Resh, 2008 ▸), as well as *O*-palmitoylation, where palmitate is irreversibly added to serine or threonine hydroxyl groups (Gao & Hannoush, 2014 ▸). No strict consensus sequence has been identified for palmitoylation; however, *S*-palmitoylated cysteine residues in yeast often exist adjacent to myristoylation or prenylation sites, and are frequently located in the cytoplasmic regions flanking, or within, transmembrane domains (Roth *et al.*, 2006 ▸; Salaun *et al.*, 2010 ▸). Palmitoylation enhances the hydrophobicity of amino acid residues, which can affect their membrane association (Rocks *et al.*, 2005 ▸). Due to the reversible nature of *S*-palmitoylation through palmitoyl acyltransferases and palmitoyl protein thioesterases, proteins have been shown to use cycles of palmitoylation/depalmitoylation to translocate intracellularly from one membrane to another (Rocks *et al.*, 2005 ▸). Palmitoylation therefore plays a significant role in protein trafficking, membrane localization and cellular signalling (Smotrys & Linder, 2004 ▸). Dysregulated palmitoylation has been shown to play a role in several human diseases, including cancer, neurological disorders and cardiovascular diseases (Ramzan *et al.*, 2023 ▸; Kong *et al.*, 2023 ▸; Baldwin *et al.*, 2023 ▸).

### 2.10. Lipidation: myristoylation

**Figure 10:**
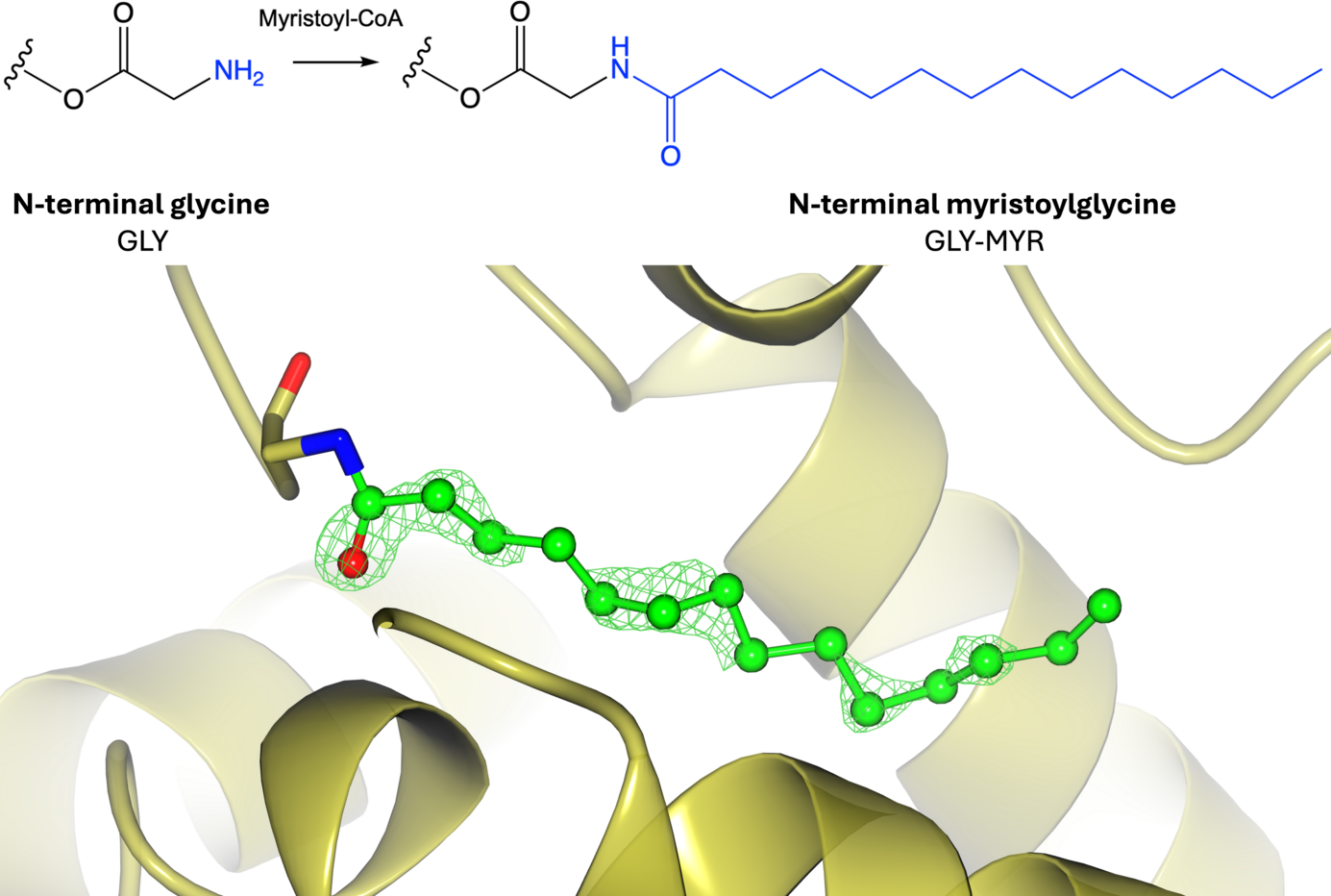
Myristoylation. Top: myristoylation of N-terminal glycine involves the addition of a myristoyl group donated by myristoyl-CoA to the N-terminal amino group. Bottom: myristoylglycine (PDB entry 4zv5; Doležal *et al.*, 2016 ▸; CCD code MYR). Positive omit density is shown in green at 3σ for the modified residue. The rest of the protein chain is represented by a yellow ribbon model.

Myristoylation involves the addition of a 14-carbon saturated fatty-acid myristoyl group to the α-amino group of an N-terminal glycine residue via an amide bond (Fig. 10 ▸; Wolven *et al.*, 1998 ▸). *N*-Myristoylated proteins generally contain the N-terminal consensus sequence M-G-*X*-*X*-*X*-S/T, where the initiator methionine is removed and the myristate is added to the exposed N-terminal glycine (Wolven *et al.*, 1998 ▸). This modification is irreversible and can be added either co-translationally or post-translationally, catalysed by *N*-myristoyltransferases (Wolven *et al.*, 1998 ▸). *N*-Myristoylation can also occur on the ɛ-amino group of internal lysine residues (Supplementary Fig. S10), although this is less common (Kosciuk *et al.*, 2020 ▸). Due to the hydrophobic nature of this modification, myristoylation plays an essential role in membrane targeting, protein–protein interactions and regulates a number of signal transduction pathways (Adam *et al.*, 2007 ▸; Hu *et al.*, 2010 ▸; Maurer-Stroh *et al.*, 2004 ▸; Timms *et al.*, 2019 ▸). However, myristoylation alone is often insufficient to stably anchor a protein to a membrane; instead a second signal is required, which involves either a cluster of hydrophobic or positively charged amino acids or a covalently attached palmitate moiety (Seykora *et al.*, 1996 ▸; Tanaka *et al.*, 1995 ▸; Gaffarogullari *et al.*, 2011 ▸). The orientation of this modification can be highly dynamic, in which the myristoylated site is either located in a hydrophobic pocket or flipped out and surface-exposed for membrane binding, which is known as a ‘myristoyl switch’ (Tanaka *et al.*, 1995 ▸). Protein myristoylation plays a role in a number of diseases, including cancer, where it has been shown to promote tumorigenesis (Tan *et al.*, 2023 ▸), as well as the virulence of HIV infections (Socas & Ambroggio, 2018 ▸; Bryant & Ratner, 1990 ▸).

### 2.11. Lipidation: *S*-prenylation

**Figure 11:**
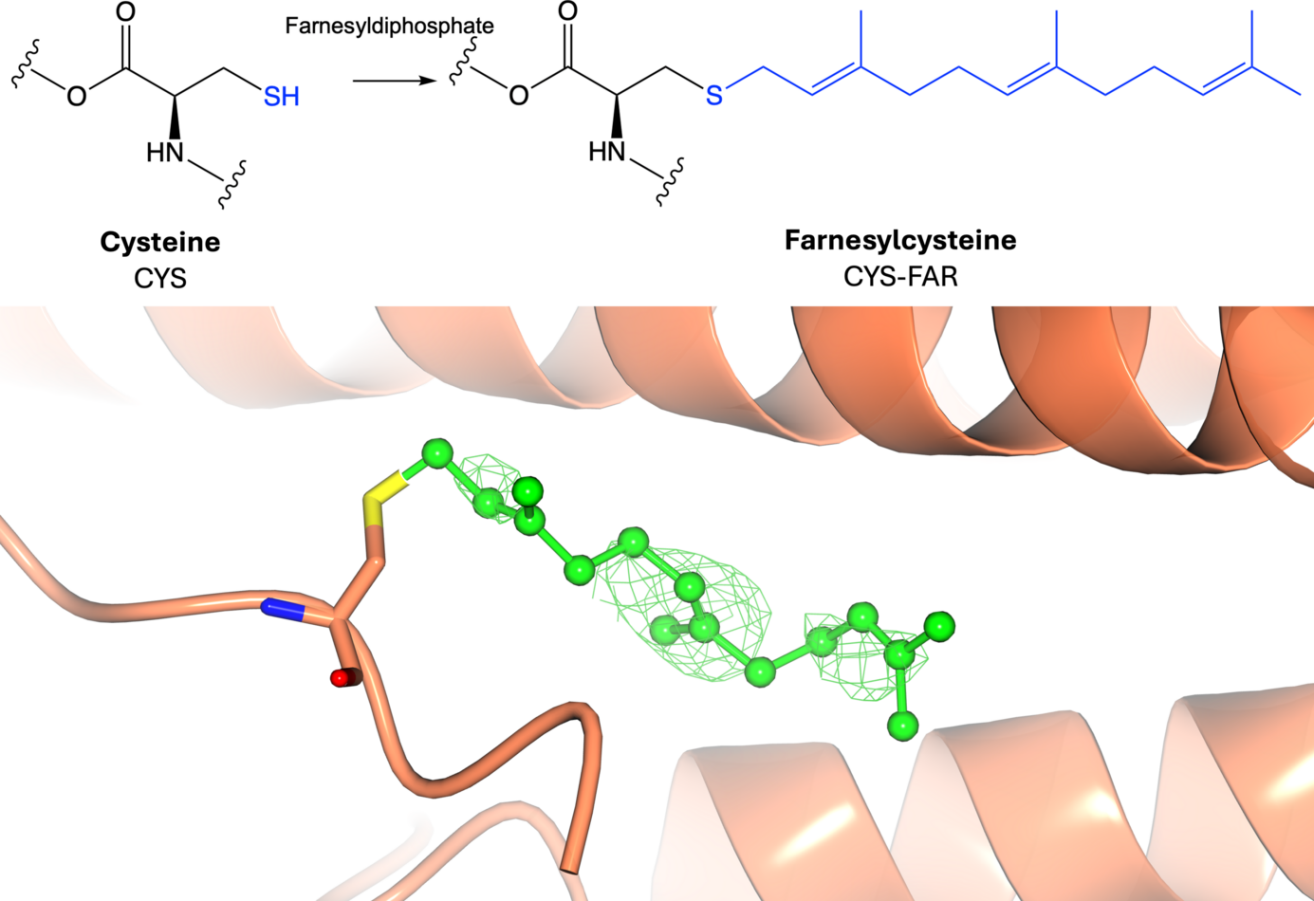
Prenylation. Top: prenylation of cysteine involves the addition of a farnesyl group (or geranylgeranyl group; Supplementary Fig. S9) donated by farnesyl diphosphate (or geranylgeranyl diphosphate) to the side-chain thiol group. Bottom: farnesylcysteine (PDB entry 6k1z; Ji *et al.*, 2019 ▸; CCD code FAR). Positive omit density is shown in green at 3σ for the modified residue. The rest of the protein chain is represented by an orange ribbon model.

Prenylation involves the irreversible addition of a 15-carbon farnesyl or a 20-carbon geranylgeranyl group to the thiol group of a cysteine residue at the carboxy-terminus via a thioester linkage (Fig. 11 ▸, Supplementary Fig. S11). A consensus motif, known as the C*AAX* box, has been identified in most prenylated proteins; in this motif, *A* stands for any aliphatic residue, while *X* represents an amino acid that determines whether the protein undergoes farnesylation or geranylgeranylation (Reid *et al.*, 2004 ▸; Seabra *et al.*, 1991 ▸). Farnesyl transferase prefers *X* to be methionine, serine, glutamine or cysteine, whereas geranylgeranyl transferase-1 prefers *X* to be leucine or isoleucine, although these rules are not absolute (Reid *et al.*, 2004 ▸; Lebowitz & Prendergast, 1998 ▸; Boutin *et al.*, 1998 ▸; Seabra *et al.*, 1991 ▸). Like palmitoylation and myristoylation, *S*-prenylation facilitates membrane association. Whilst geranylgeranylation is sufficiently hydrophobic to facilitate membrane anchoring, farnesylated proteins require a second signal for stable membrane interaction, typically palmitoylation or a cluster of positive amino acids (Cuiffo & Ren, 2010 ▸; Hancock *et al.*, 1990 ▸). Protein prenylation has implications in diseases such as cancer and diabetes (Borini Etichetti *et al.*, 2020 ▸; Gendaszewska-Darmach *et al.*, 2021 ▸).

## 3. PTMs in the Protein Data Bank

### 3.1. PTM annotation in macromolecular crystallographic files

PTMs have versatile annotations in macromolecular crystallographic files that are dependent on the type of PTM. Small-molecule PTMs are defined as modifications that include ten or fewer atoms (for example phosphorylation, hydroxylation and methylation), whereas long-chain PTMs include greater than ten atoms (glycosylation and lipidation) (wwPDB Processing Procedures and Policies Document, Section A, 2014 ▸). For small-molecule PTMs, both the modification and the residue atoms are listed within the same three-letter CCD code and are part of the polymeric sequence of the protein. For example, *SEP* includes both the atoms of the serine residue and the phosphate group involved in the modification. In contrast, for protein glycosylation and most cases of lipidation, the CCD code does not include the residue atoms; instead the modification is covalently linked to the polymer. For example, *FAR* includes atoms of a farnesyl group and does not include the atoms of the cysteine residue to which it is linked.

Within legacy PDB and mmCIF files, the CCD codes of small-molecule PTMs are listed in the modified residue data items (PDB, MODRES; mmCIF, _pdbx_struct_mod_residue), which include any modified polymer components. For protein glycosylation, the modified amino acid residue is also listed here, *e.g.ASN*, *SER*, *THR* are listed, without the sugar. In mmCIF files, protein glycosylation is clearly annotated due to unique data items for glycans. Firstly, _pdbx_entity_branch_link contains the sugar-chain information, and secondly _struct_conn.pdbx_role, contains information regarding the glycan type, with allowed values of *C*-mannosylation, *N*-glycosylation, *O*-glycosylation or *S*-glycosylation. Annotation in crystallographic files differs for lipids; in mmCIF files, lipid CCD codes can be found in a number of data items that highlight special features or structurally relevant sites, including _struct_site, _struct_site_gen and _pdbx_entity_instance_feature, as well as in _pdbx_entity_nonpoly and pdbx_nonpoly_scheme, which provide information about nonpolymeric components.

PTMs therefore have versatile annotations in macromolecular crystallographic files, with no specific data item dedicated to PTMs, apart from for glycosylation. Even for small-molecule PTMs, the modified-residue data category includes any modified polymer components, which can include coenzymes and synthetic chemical modifications (such as chromophores). Furthermore, there are issues with redundant labelling in lipidation. While most cases are listed as covalently linked to a polymer, there are instances where lipid modifications are annotated similarly to small-molecule PTMs, with both the lipid molecule and residue atoms included within the CCD code. For example, lysine myristoylation is represented by *MYK*, which includes the atoms for the lysine residue and the myristoyl group and is found in the modified-residue category.

### 3.2. Frequency of PTMs in the PDB

Currently, there is no single data item in mmCIF files that allows for exclusive searches of PTMs in the PDB. As a result, this analysis used individual CCD codes to search for PTMs in the PDB. Initially, a list of PTM-related keywords was constructed using the UniProtKB PTM list, which describes the chemical nature of protein modifications using a controlled vocabulary (UniProt Consortium, 2021 ▸); this includes annotations of both RESID (Reference Sequence Identifier; Garavelli, 2004 ▸) and CHEBI (Chemical Entities of Biological Interest; Hastings *et al.*, 2016 ▸) identifiers. Entries were removed if they were not defined as a PTM by comparison with the dbPTM, although analysis was still performed (Supplementary Fig. S12). Following this, PTMs were matched to their CCD codes, primarily using a sub­structure search between the CCD and ChEBI databases, using *RDKit* (https://www.rdkit.org). In addition, the RESID was used to search the PDB via the RCSB PDB Search API (Bittrich *et al.*, 2023 ▸; Rose *et al.*, 2021 ▸) to generate a dataset of individual RESIDs and associated protein structures; subsequently, a compilation of covalently linked CCD codes was identified and linked to each RESID. This allowed the generation of an exhaustive PTM list with corresponding CCD codes, which was then used to search the PDB via the RCSB PDB Search API (Bittrich *et al.*, 2023 ▸; Rose *et al.*, 2021 ▸).

**Figure 12:**
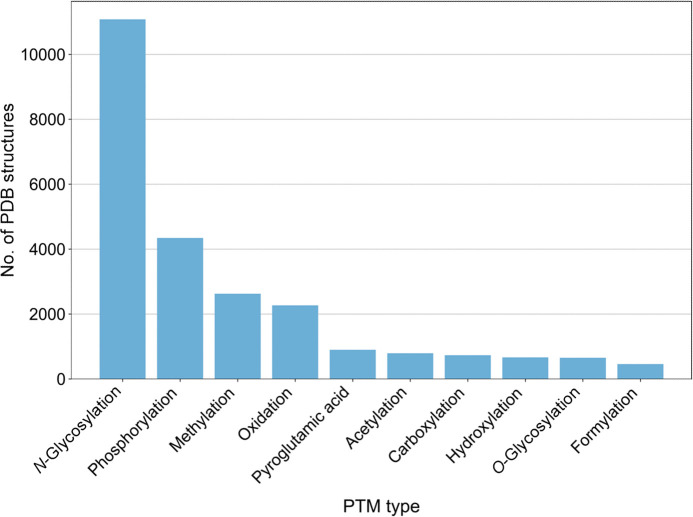
Most common types of PTM in the PDB. The top ten PTM types identified in the PDB are shown. Data were obtained by searching the RCSB PDB Search API using identified CCD codes corresponding to PTMs. Glycosylation data were obtained from the *Privateer* Database (Dialpuri, Bagdonas, Schofield, Pham, Holland, Bond *et al.*, 2024 ▸; Dialpuri, Bagdonas, Schofield, Pham, Holland & Agirre, 2024 ▸).

**Figure 13:**
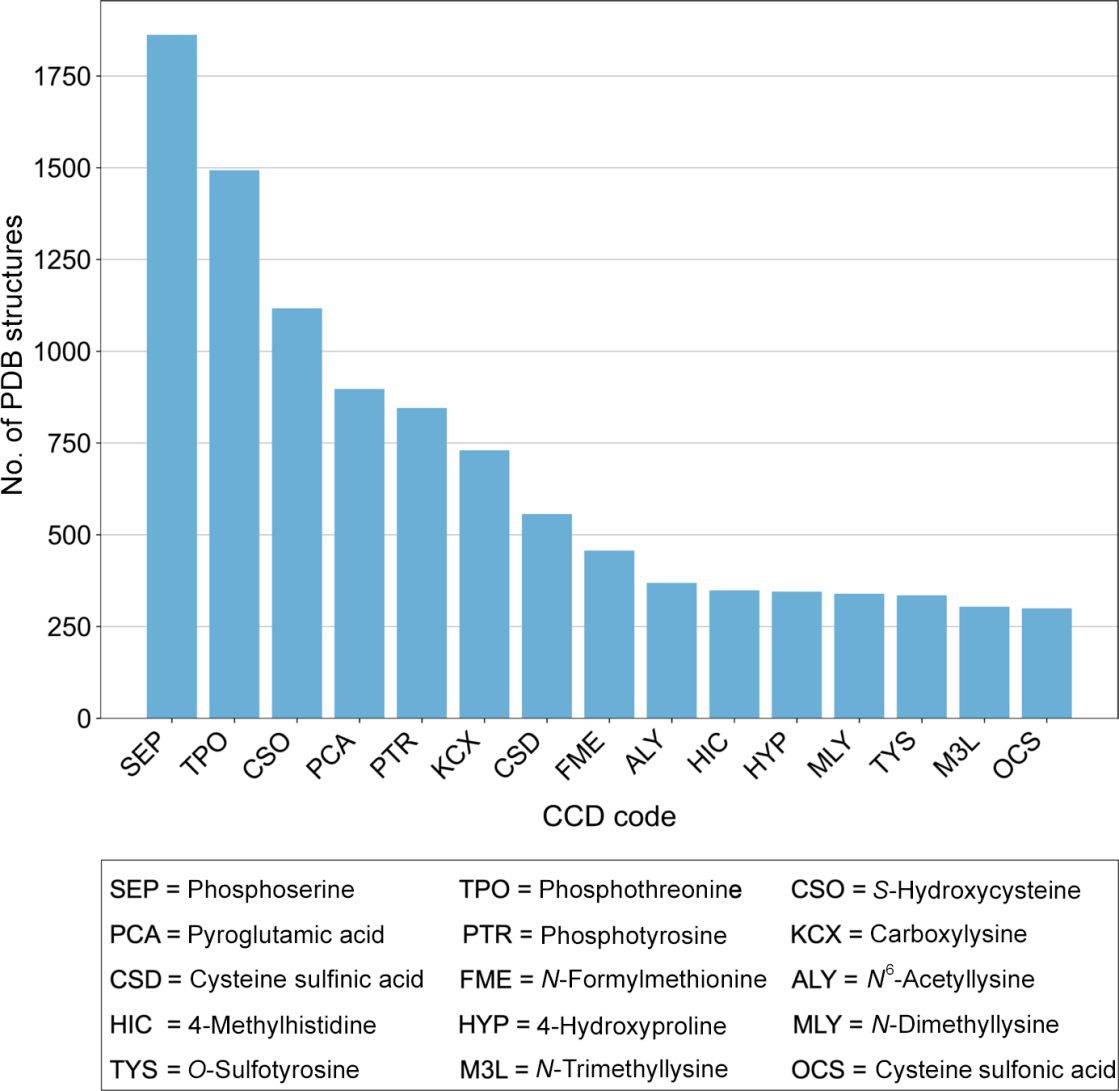
Most common small-molecule post-translationally modified residues in the PDB. The top 15 small-molecule PTMs identified in the PDB are shown. Data were obtained by searching the RCSB PDB Search API using identified CCD codes corresponding to PTMs. These data include CCD codes which are located in the polymeric sequence.

In total, over 23 000 examples of PDB entries containing post-translationally modified residues were identified. The most common PTM type identified was *N*-glycosylation, which makes up nearly half of the identified PTMs (Fig. 12 ▸). This is followed by phosphorylation, methylation and acetylation. Generally, these trends align well with the number of PTM sites reported in the dbPTM (Table 1 ▸), with *N*-glycosylation and phosphorylation being the most well studied. In fact, phosphoserine, phosphotyrosine and phosphothreonine are among the most common small-molecule PTMs identified in the PDB (Fig. 13 ▸). Methylation and hydroxylation modifications were also identified as some of the most common PTM types in the PDB, with dimethyllysine, trimiethyllysine, acetyllysine and hydroxyproline amongst the most common small-molecule PTMs to be identified. In addition, oxidation has been identified as a common PTM in the PDB, with hydroxycysteine, cysteine sulfinic acid and cysteine sulfonic acid in high abundance. When analysing protein oxidation in the context of crystallography, it is key to recognize that radiation damage can promote cysteine oxidation, meaning that oxidized cysteines may not always be biologically relevant (Close & Bernhard, 2019 ▸; Garrido Ruiz *et al.*, 2022 ▸). Furthermore, there is a prevalence of acetylation, hydroxylation and *O*-glycosylation, further highlighting these modifications as some of the most well studied, as suggested in the dbPTM (Table 1 ▸).

Whilst pyroglutamic acid, carboxylation and formylation were identified as common PTM types in the PDB, they are less common both putatively and experimentally as reported in the dbPTM (Li *et al.*, 2022 ▸). For pyroglutamic acid, this may be explained by the spontaneous formation of pyroglutamic acid *in vitro*, where its formation has been attributed to being an artefact during sample preparation and storage of immunoglobulins, which may account for the increased occurrence of this modification in the PDB (Chelius *et al.*, 2006 ▸). Furthermore, the extent of lysine carboxylation, a spontaneous PTM, is not fully understood; however, it has been found in a number of key enzymes, including pyruvate carboxylase, urease, RuBisCo and β-lactamase (Jimenez-Morales *et al.*, 2014 ▸; Sheng *et al.*, 2019 ▸). Furthermore, *N*-formylmethionine is an amino acid derivative that is key for prokaryotic protein synthesis; it is encoded by the AUG codon, which is the start codon for protein synthesis. It is therefore the N-terminal amino acid of nearly all proteins in prokaryotic systems, which explains its prevalence in the PDB. As a result, it is typically considered to be a pre-translational modification rather than a PTM. Whilst these results are interesting, it is key to consider that as the PDB was used as the primary data source, redundant depositions may also explain the prevalence of less common PTMs.

**Figure 14:**
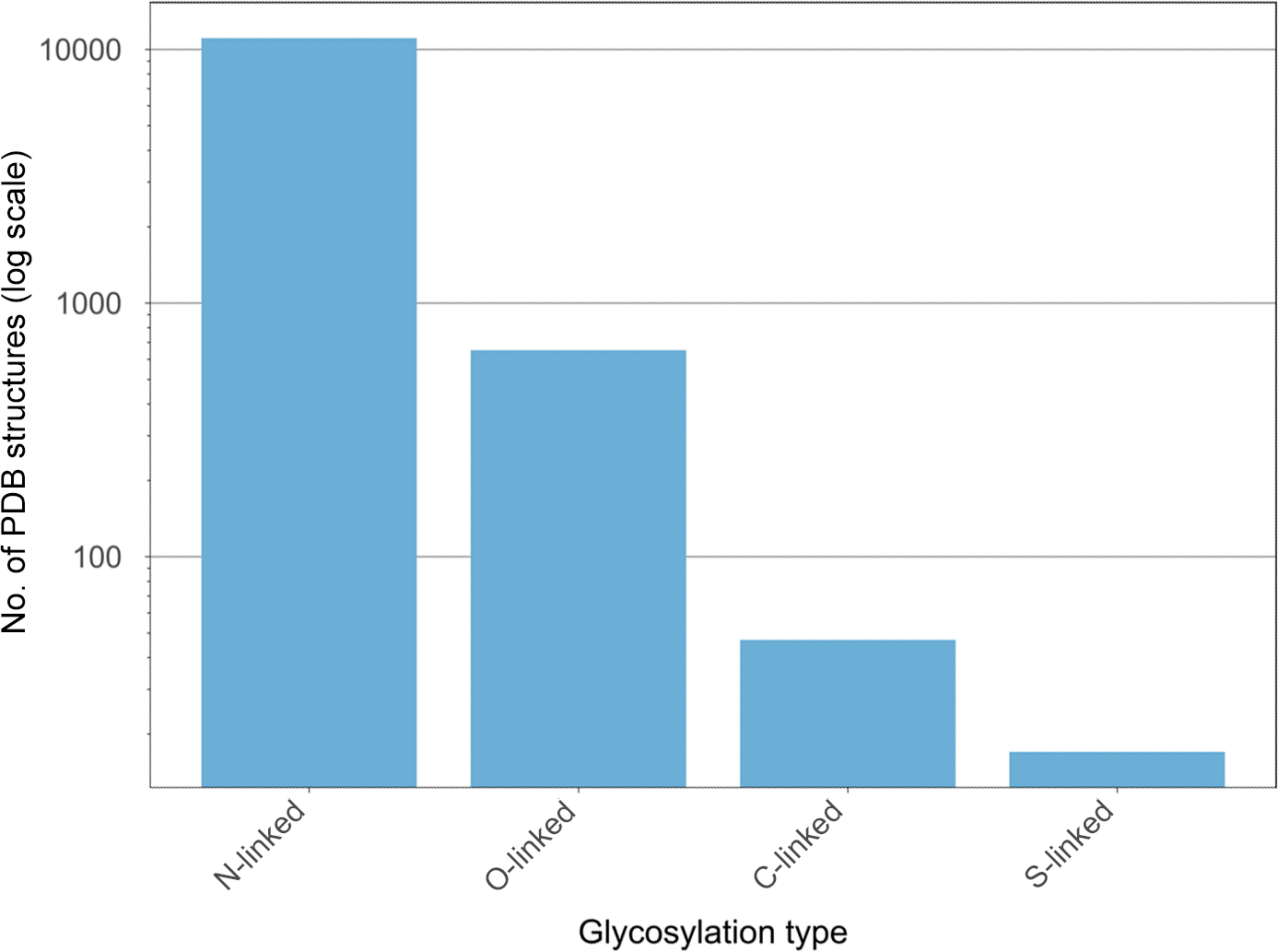
Glycosylation types in the PDB. Bars show the number of PDB structures containing each glycosylation type. The *y* axis is shown in log count. Data were obtained from the *Privateer* database (Dialpuri, Bagdonas, Schofield, Pham, Holland & Agirre, 2024 ▸; Dialpuri, Bagdonas, Schofield, Pham, Holland, Bond *et al.*, 2024 ▸).

**Figure 15:**
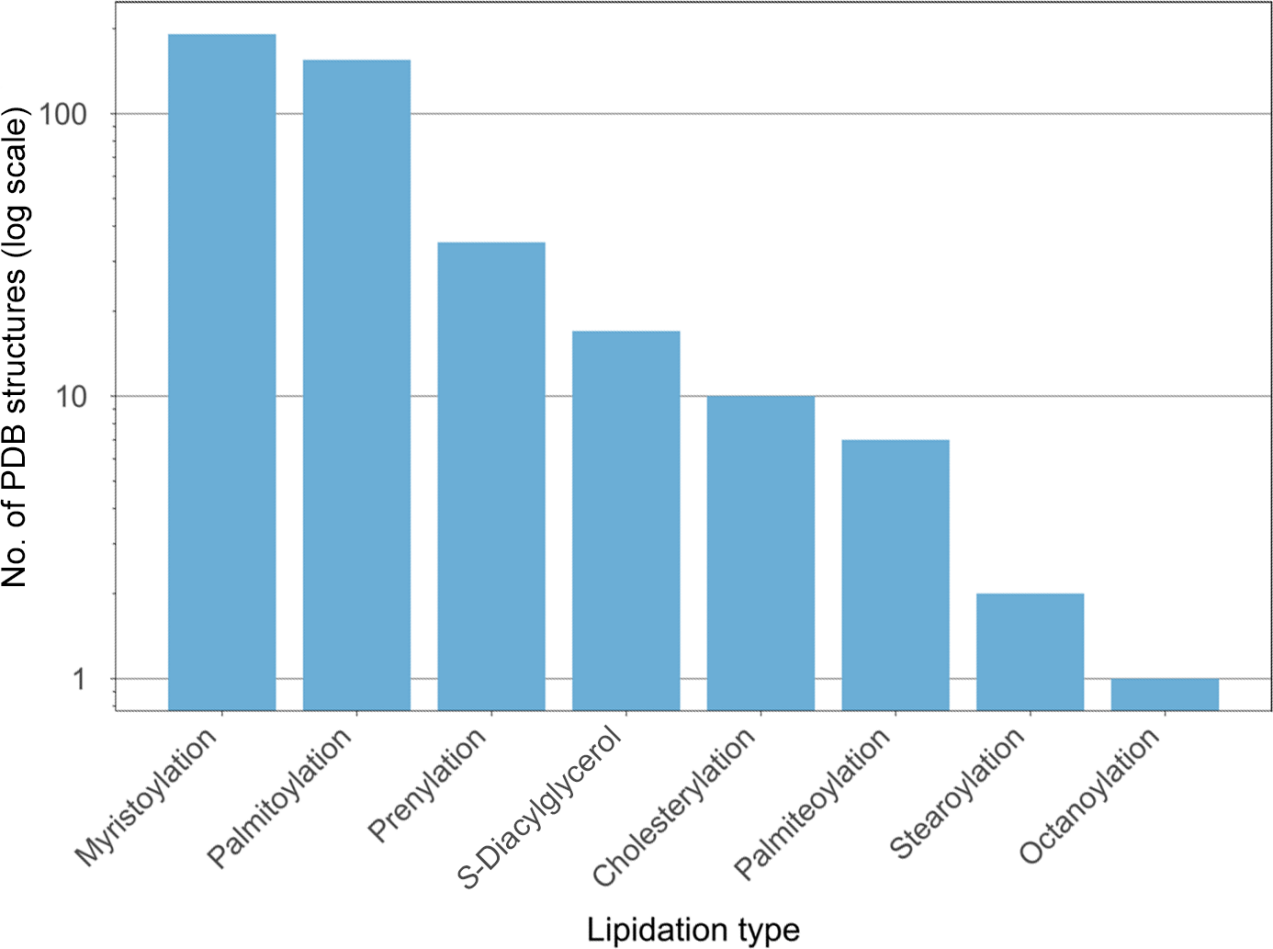
Lipidation types in the PDB. Bars show the number of PDB structures containing each detected lipidation type. The *y* axis is shown in log count. Data were obtained by searching the RCSB PDB Search API using identified CCD codes corresponding to lipid PTMs.

It is well known that *N*-glycans are the most frequent type of glycosylation, followed by *O*-glycans, with fewer examples of *C*- and *S*-glycans (Fig. 14 ▸), and the trends shown in the PDB seem to reflect this well. Whilst glycans are often hard to deal with in crystallography, *Privateer* (Agirre *et al.*, 2015 ▸; Dialpuri, Bagdonas, Schofield, Pham, Holland, Bond *et al.*, 2024 ▸), a software package that identifies and rectifies model errors in protein–glycan structures, has facilitated the modelling of sugars. *Privateer* is able to produce torsion restraints (Atanasova *et al.*, 2022 ▸), as well as linkage torsion analysis (Dialpuri *et al.*, 2023 ▸), allowing refinement to produce more accurate and representative models with ease. Lipidation PTMs, however, were identified less frequently in the PDB, likely due to their less ordered, flexible structures that can lead to partial occupancy (Fig. 15 ▸). Myristoylation and palmitoylation are the most common forms of lipidation identified in the PDB, reflecting well the most common putative and experimental lipidation sites reported in the dbPTM (Table 1 ▸).

**Figure 16:**
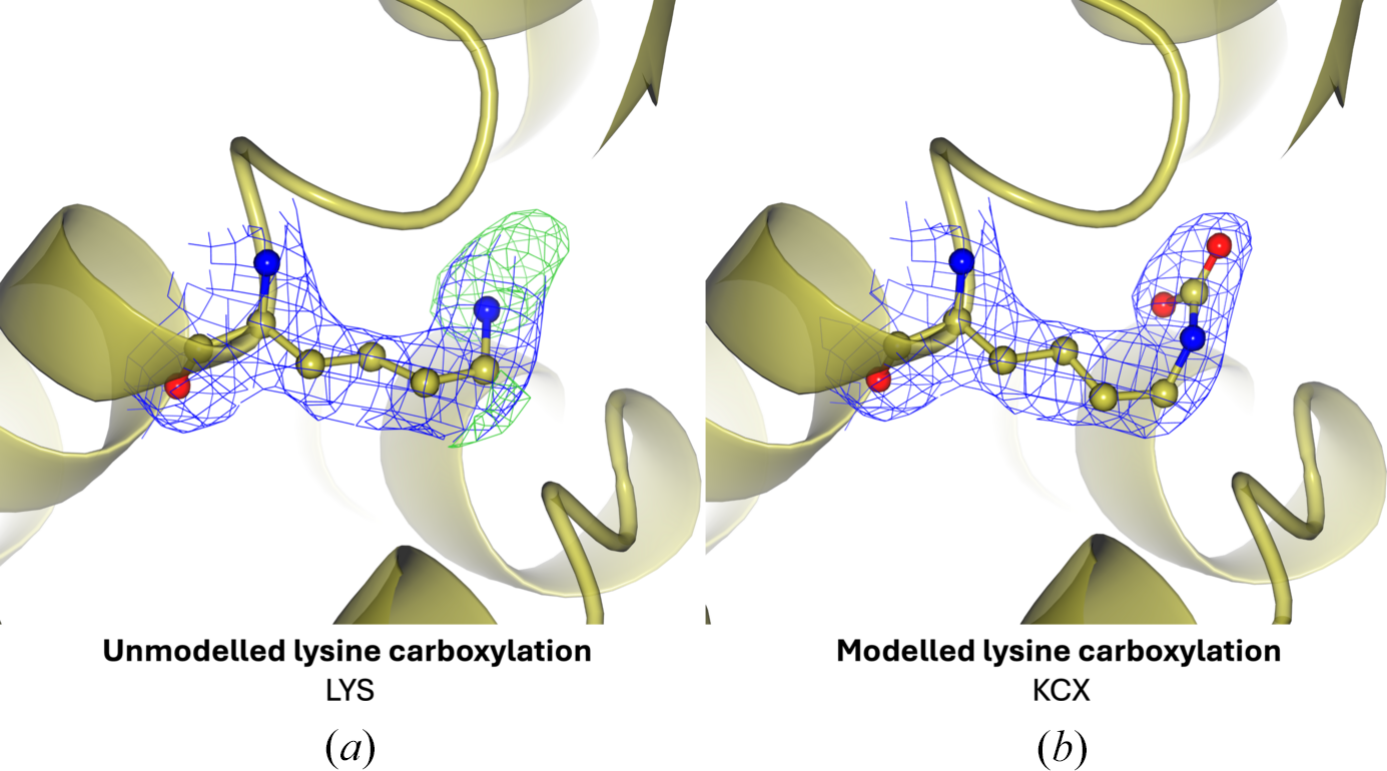
Unmodelled PTMs in the PDB. Density for protein modifications may be present but left unmodelled. (*a*) Lys84 has positive difference density surrounding the side-chain amino group (PDB entry 2jc7; Santillana *et al.*, 2007 ▸). (*b*) Addition of a carboxyl group to the NZ atom of Lys84 [using *Coot* (Emsley *et al.*, 2010 ▸) and *AceDRG* (Long *et al.*, 2017 ▸)], followed by refinement with *REFMAC* (Murshudov *et al.*, 2011 ▸). 2*mF*_o_ − *DF*_c_ electron density is shown in blue at 1σ for the residue. Positive difference density (*mF*_o_ − *DF*_c_) is shown in green contoured to 4σ and clipped within 4 Å of Lys84. The rest of the protein chain is represented by a yellow ribbon model.

One factor that should be considered when analysing PTMs in the PDB is the difficulty of searching for isopeptides, a PTM which involves the formation of bonds between amino acid side chains, which are not well annotated in PDB files and have therefore not been captured in this analysis. Another factor that should be considered when analysing PTMs in the PDB is the possibility that protein modifications have been captured by experimental techniques but have not been modelled. *PreLysCar*, a prediction tool for lysine carboxylation, highlights an example (Fig. 16 ▸) where a lysine residue is predicted to be carboxylated (Jimenez-Morales *et al.*, 2014 ▸). Having inspected the difference density (*mF*_o_ − *DF*_c_) surrounding the lysine residue, a region of positive difference density surrounding the NZ atom can be identified, into which a carboxyl group can be modelled (Jimenez-Morales *et al.*, 2014 ▸). This highlights the idea that there may be unmodelled PTMs in the PDB, where there is density present but they have not been modelled. This suggests that this analysis is almost certainly an underestimation of the number of PTMs in the PDB.

## 4. Challenges and future directions

Having understood the importance of PTMs in protein structure dynamics, it is clear that they are essential for the understanding of protein structure and function. It is key to recognize the importance of integrating structural data with functional and biochemical data, which provides a comprehensive understanding of how PTMs regulate protein function at the molecular level. This will aid in the understanding of the mechanisms underlying PTM-mediated regulation, which is key for many biochemical processes. A significant challenge facing PTMs in protein structures is the lack of consistent, exclusive annotation within macromolecular structure files. In this study, identifying PTMs in the PDB was primarily performed using a substructure search using *RDKit* (https://www.rdkit.org). Whilst this was effective, some PTMs may have been missed. Fortunately, the wwPDB is in the process of standardizing PTM annotation in mmCIF files for both PDB and CCD entries, making it easier to search for all PTM types by the beginning of 2025 (wwPDB, 2024 ▸).

Going forward, several advancements in protein structure-determination techniques will aid in the investigation of PTMs. We can now capture heterogeneous protein populations, which is particularly useful in the study of PTMs. Specifically, this can be performed using methods such as cryo-EM SPA, which allows the reconstruction of protein structures from thousands of individual images, as well as serial femtosecond crystallography, which allows diffraction patterns to be obtained for individual molecules. As techniques for protein structure determination improve, it is important to develop semi-automatic tools for PTM identification, modelling and validation. This will allow users to edit their structures quickly and will speed up a once time-consuming task. In conclusion, while there are several challenges in the study of PTMs at the structural level, advancements in structure-determination techniques, the improvement of PTM annotation in PDBx/mmCIF model files, and the increasing awareness of their importance will facilitate the study of PTMs going forward.

## 5. Related literature

The following references are cited in the supporting information for this article: Bokhovchuk *et al.* (2023 ▸), Ferrara *et al.* (2011 ▸), Gokulan *et al.* (2013 ▸), Hilgers & Ludwig (2001 ▸), Huang *et al.* (2023 ▸), Kumar *et al.* (2022 ▸), Lee & Paetzel (2011 ▸), Merő *et al.* (2019 ▸), Oakley *et al.* (2002 ▸) and Yang *et al.* (2013 ▸).

## Supplementary Material

Supplementary Figures. DOI: 10.1107/S2059798324007794/pea5001sup1.pdf

## Figures and Tables

**Table 1:** The top 15 PTM sites listed in the dbPTM The PTMs with the highest number of total sites are listed. As of February 2024, there were 542 107 putative sites and 2 235 664 experimentally determined PTM sites in the dbPTM (Li *et al.*, 2022 ▸). Due to a lack of data in the dbPTM, experimental data for 2-hydroxyisobutyrlation was taken from *DeepKhib*, which used experimentally determined sites to train the predictor algorithm (Zhang *et al.*, 2020 ▸).

Modification type	No. of experimental sites	No. of putative sites	Total sites
Phosphorylation	1615150	160490	1775640
Ubiquitination	348308	108349	456657
Acetylation	138171	38530	176701
*N*-Linked glycosylation	27366	89143	116509
Methylation	16114	16766	32880
2-Hydroxyisobutyrylation	12166	32392	44558
*O*-Linked glycosylation	16696	8809	25505
Succinylation	17973	6387	24360
Malonylation	12847	145	12992
Sumoylation	5889	5731	11620
*S*-Palmitoylation	6505	3409	9914
Sulfoxidation	7581	0	7581
Hydroxylation	2404	4543	6947
Amidation	3316	1462	4778
*S*-Nitrosylation	4172	483	4655
