# Supplementary material for: Post-translational modifications in the Protein Data Bank
Source: Acta Crystallogr D Struct Biol. 2024 Aug 29;80(Pt 9):647–60. doi: 10.1107/S2059798324007794 (PMC11394121; doi:10.1107/S2059798324007794)
Supplement: Supplementary file 1 [file d-80-00647-sup1.pdf]

STRUCTURAL
BIOLOGY

Volume 80 (2024)

Supporting information for article:

Post-translational modifications in the Protein Data Bank

Lucy C. Schofield, Jordan S. Dialpuri, Garib N. Murshudov and Jon Agirre

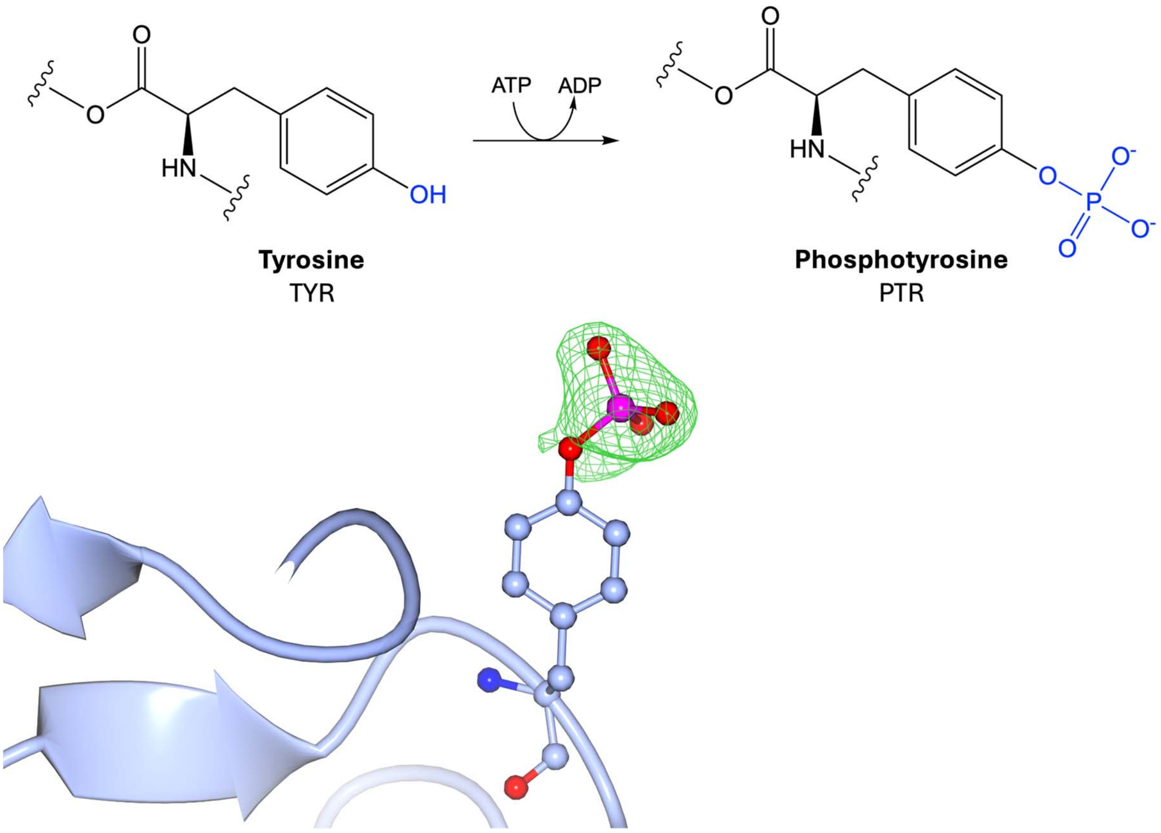

Figure S1 Phosphorylation. (Top panel) Phosphorylation of tyrosine involves the addition of a phosphate group donated by ATP to the side chain hydroxyl group. (Bottom panel) Phosphotyrosine (PDB code: 5NP2 (Merő et al., 2019), CCD code: PTR). Positive omit density is shown in green at 3σ for the modified residue. The rest of the protein chain is represented by a blue ribbon model.

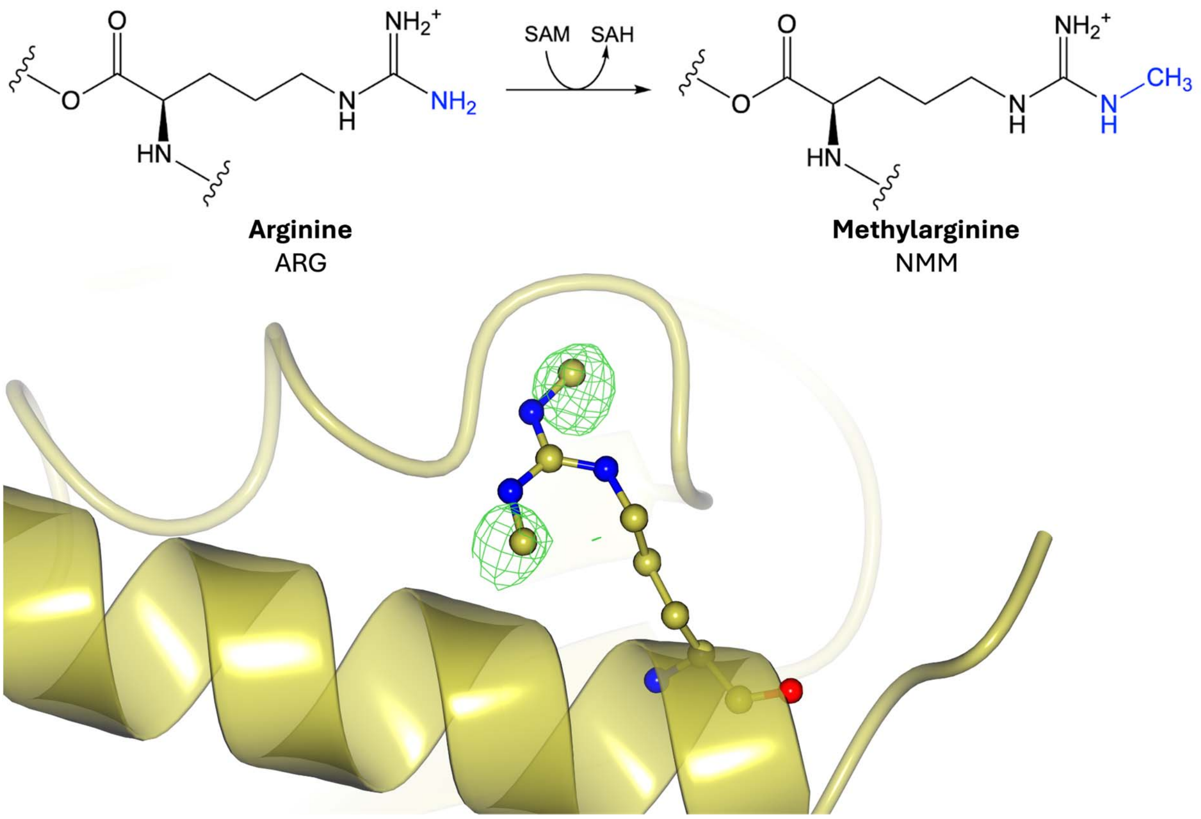

Figure S2 Methylation. (Top panel) Methylation of arginine involves the addition of a methyl group donated by S-Adenosyl methionine to the side chain guanidino group. (Bottom panel) Methylarginine (PDB code: 1G42 (Oakley et al., 2002), CCD code: NMM). Positive omit density is shown in green at 3σ for the modified residue. The rest of the protein chain is represented by a yellow ribbon model.

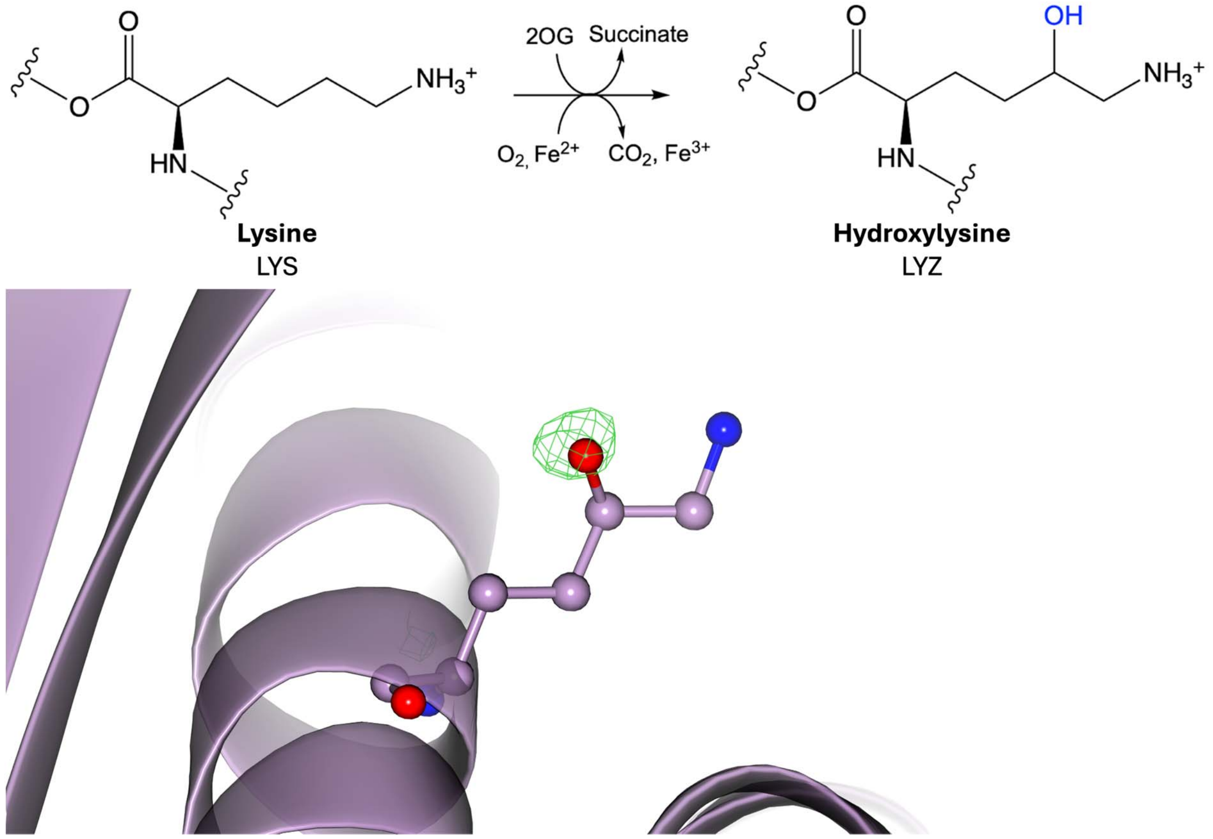

Figure S3 Hydroxylation. (Top panel) Hydroxylation of lysine involves the addition of a hydroxyl group donated by 2-oxoglutarate (2OG) to the side chain amino group. (Bottom panel) Hydroxylysine (PDB code: 6KHH (Kumar et al., 2022), CCD code: LYZ). Positive omit density is shown in green at 3σ for the modified residue. The rest of the protein chain is represented by a purple ribbon model.

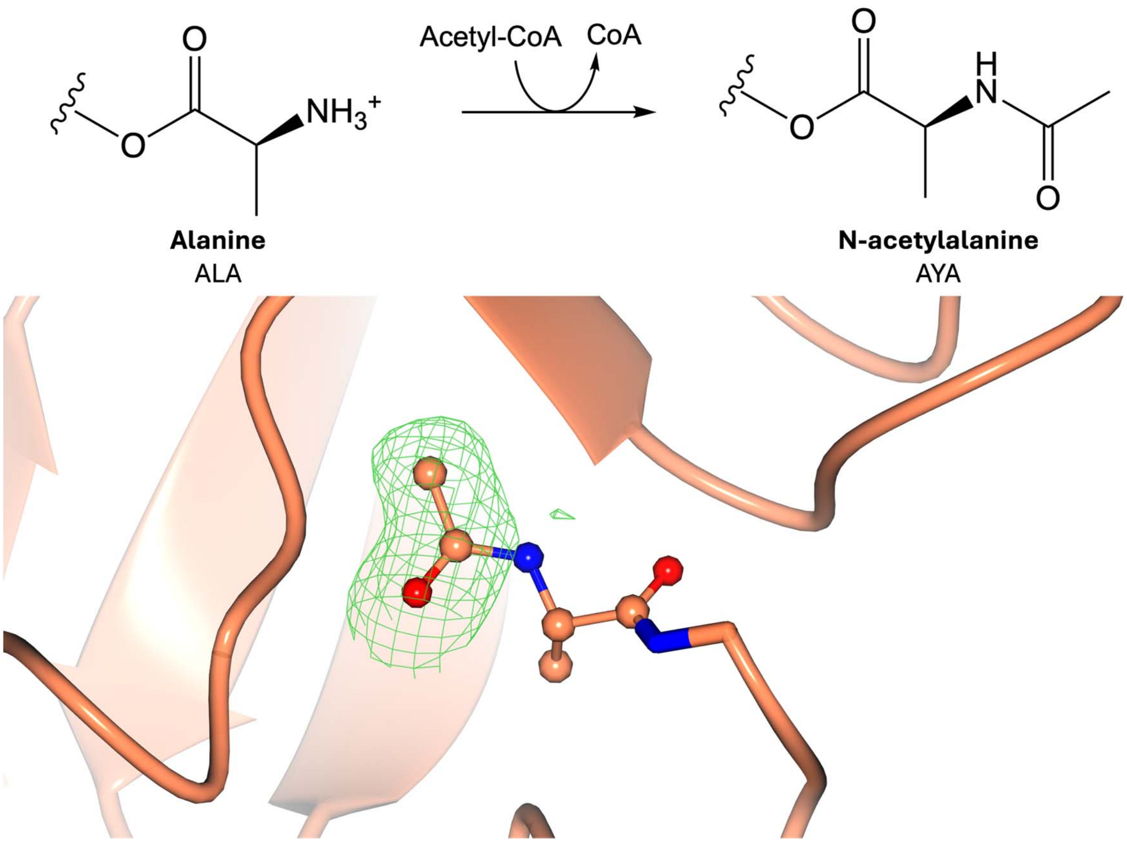

Figure S4 Acetylation. (Top panel) N-terminal acetylation of alanine involves the addition of an acetyl group donated by acetyl-CoA to the N-terminal amino group. (Bottom panel) Acetylalanine (PDB code: 4KUI (Yang et al., 2013), CCD code: AYA). Positive omit density is shown in green at 3σ for the modified residue. The rest of the protein chain is represented by an orange ribbon model.

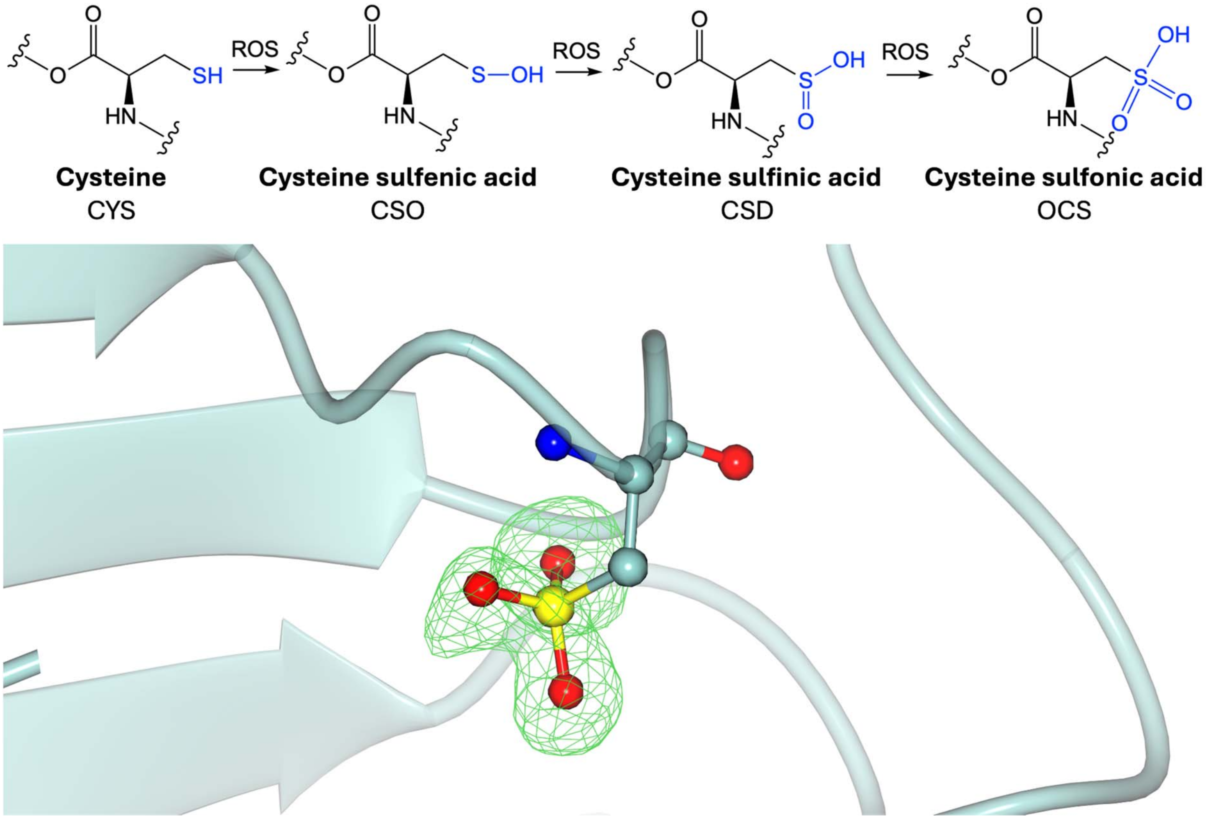

Figure S5 Oxidation. (Top panel) Oxidation of cysteine involves the reaction between the side chain thiol group of cysteine and reactive oxygen species (ROS) to form cysteine sulfenic, then cysteine sulfinic acid, and cysteine sulfonic acid. (Bottom panel) Cysteine sulfonic acid (PDB code: 1IE0 (Hilgers & Ludwig, 2001), CCD code: OCS). Positive omit density is shown in green at 3σ for the modified residue. The rest of the protein chain is represented by a blue ribbon model.

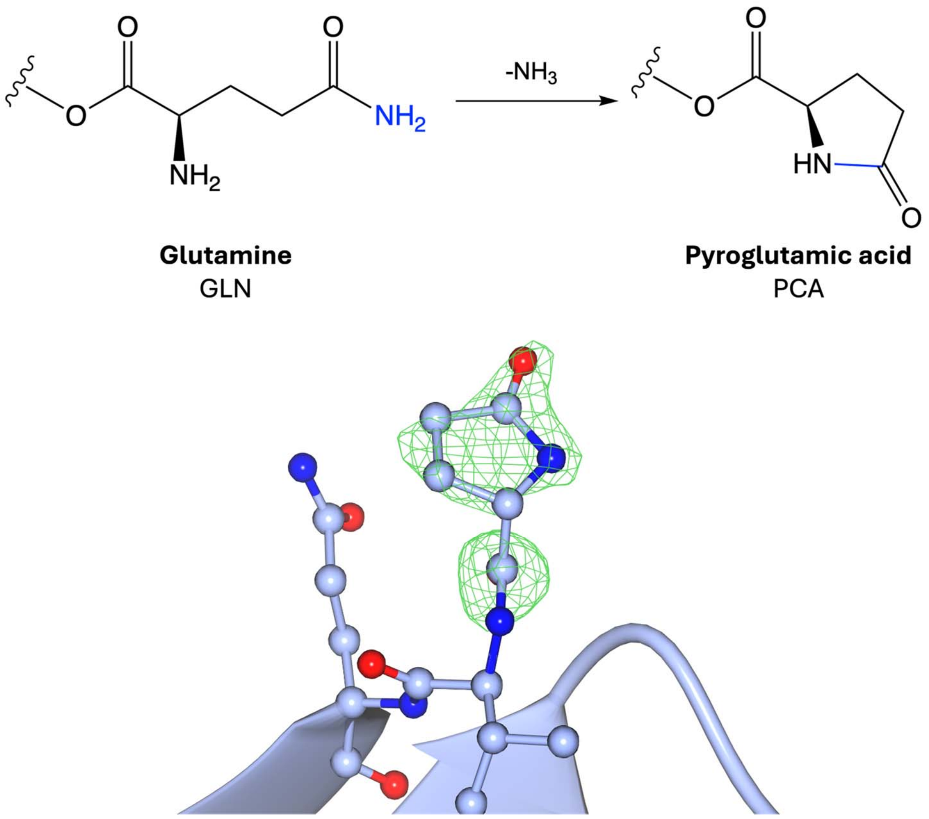

Figure S6 Pyroglutamic acid. (Top panel) Formation of pyroglutamic acid involves the cyclisation of an N-terminal glutamine or glutamic acid. (Bottom panel) Pyroglutamic acid (PDB code: 8ECV (Huang et al., 2023), CCD code: PCA). The first three N-terminal residues are shown. Positive omit density is shown in green at 3σ for the modified residue. The rest of the protein chain is represented by a blue ribbon model.

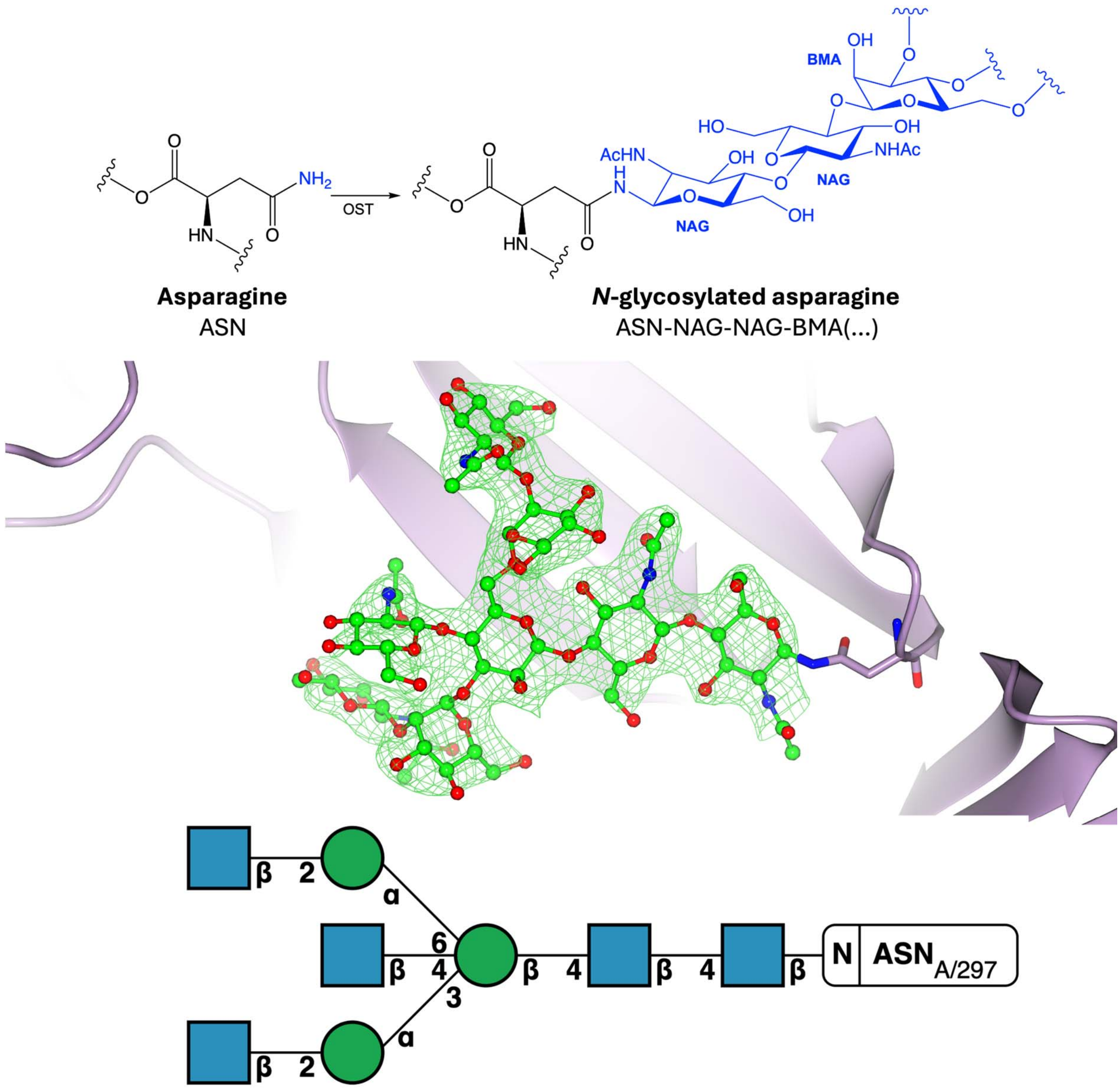

Figure S7 N-glycosylation. (Top panel) N-glycosylation of asparagine involves the addition of an N-glycan to the side chain amino group via oligosaccharide transferase (OST). (Middle panel) N-linked glycosylation (PDB: 3SGK (Ferrara et al., 2011)). Positive omit density is shown in green at 3σ for the modified residue. The rest of the protein chain is represented by a purple ribbon model. (Bottom panel) The symbol nomenclature for glycans (SNFG) representation is shown and was generated using the Privateer WebApp (Dialpuri, Bagdonas, Schofield, Pham, Holland, Bond et al., 2024).

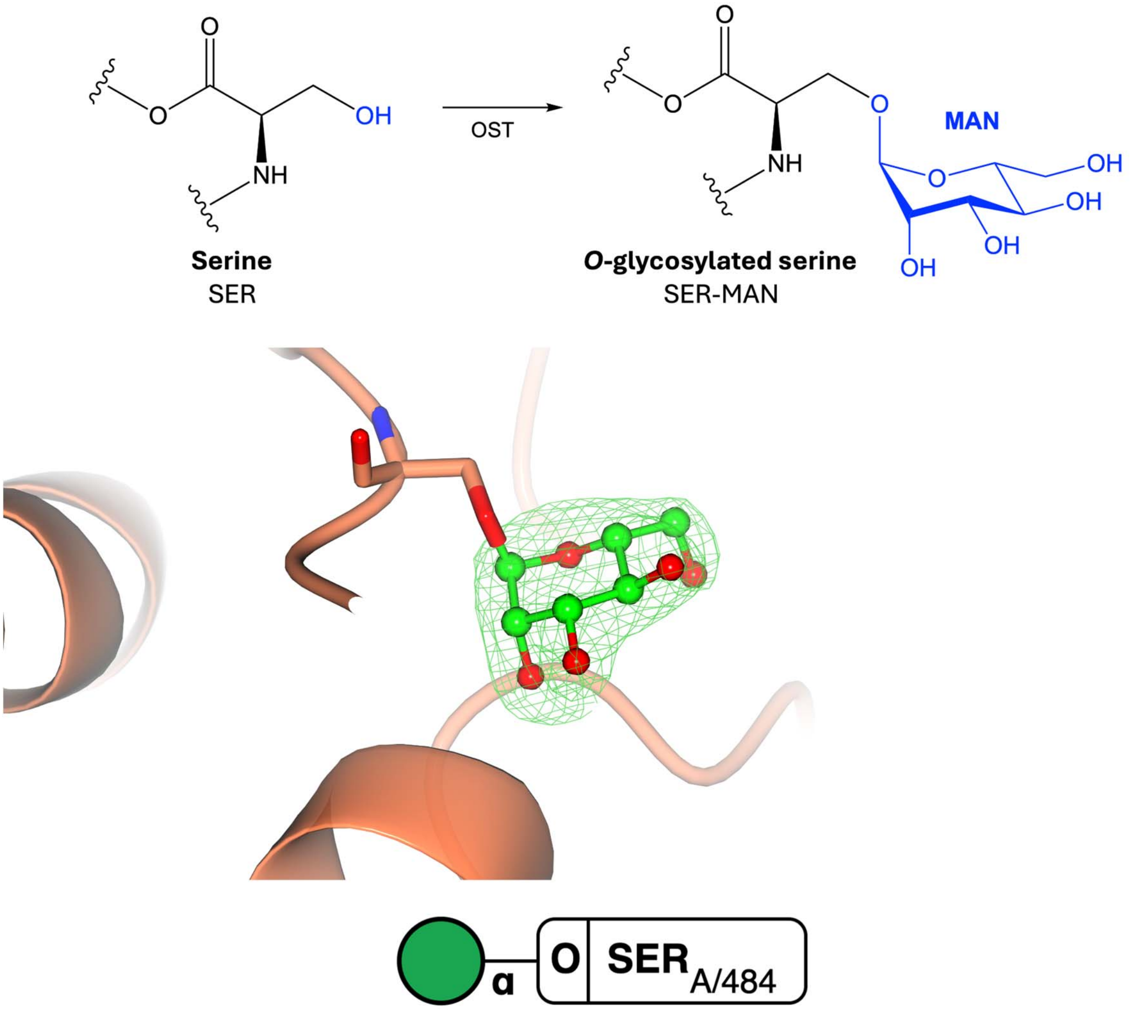

Figure S8 O-glycosylation. (Top panel) O-glycosylation of serine or threonine involves the addition of an O-glycan to the side chain hydroxyl group via oligosaccharide transferase (OST). (Middle panel) O-linked glycosylation (PDB code: 3EQA (Lee & Paetzel, 2011)). Positive omit density is shown in green at 3σ for the modified residue. The rest of the protein chain is represented by an orange ribbon model. (Bottom panel) The symbol nomenclature for glycans (SNFG) representation is shown and was generated using the Privateer WebApp (Dialpuri, Bagdonas, Schofield, Pham, Holland, Bond et al., 2024).

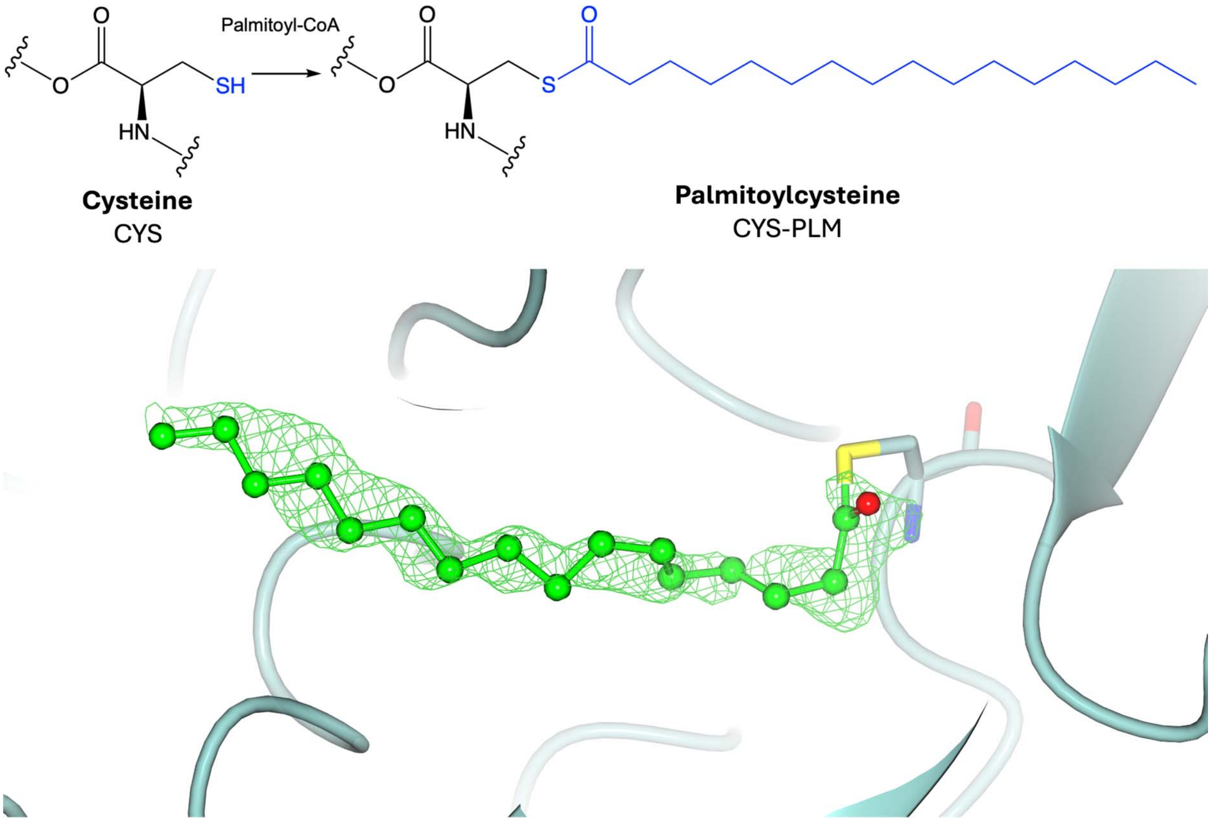

Figure S9 Palmitoylation. (Top panel) Palmitoylation of cysteine involves the addition of a palmitoyl group donated by palmitoyl-CoA to the side chain thiol group. (Bottom panel) Palmitoylcysteine (PDB code: 4JAQ (Gokulan et al., 2013), CCD code: PLM). Positive omit density is shown in green at 3σ for the modified residue. The rest of the protein chain is represented by a blue ribbon model.

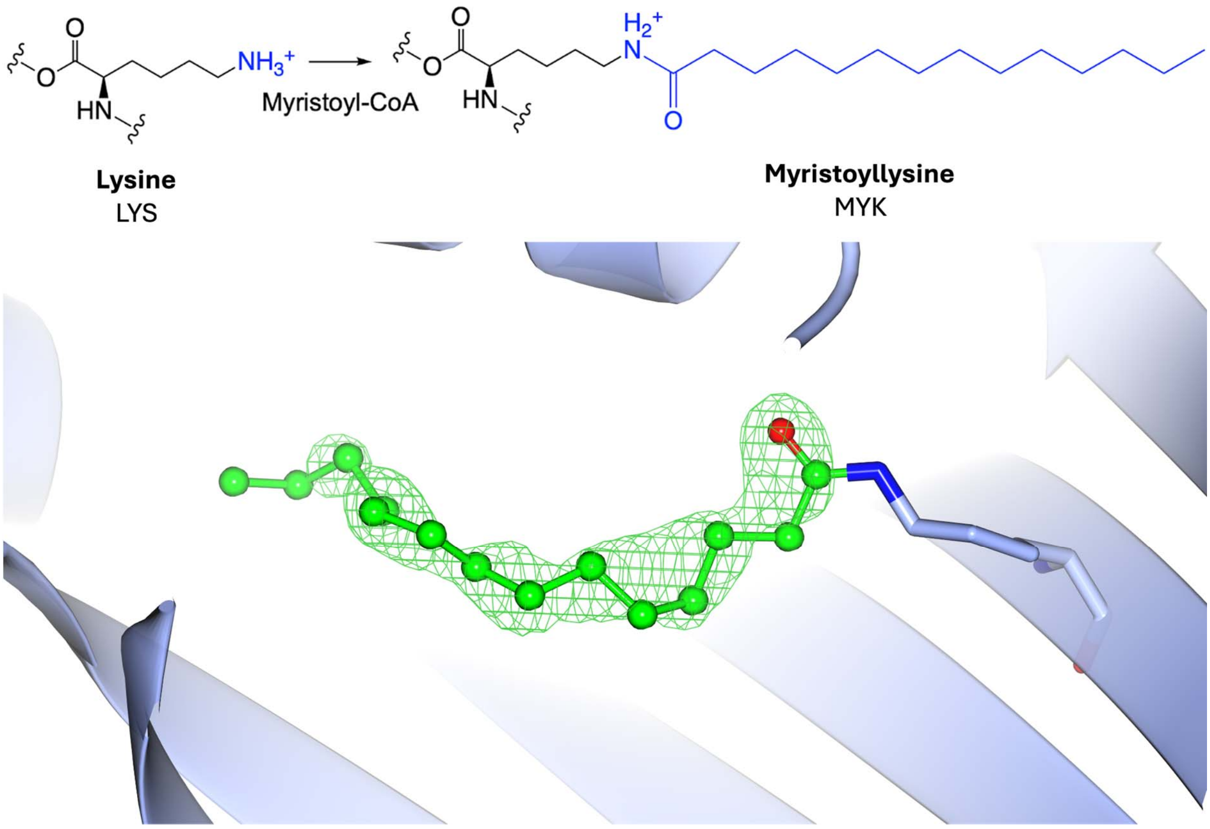

Figure S10 Myristoylation. (Top panel) Myristoylation of lysine involves the addition of a myristoyl group donated by myristoyl-CoA to the side chain amino group. (Bottom panel) Myristoyllysine (PDB code: 8A8Q (Bokhovchuk et al., 2023), CCD code: MYK). Positive omit density is shown in green at 3σ for the modified residue. The rest of the protein chain is represented by a blue ribbon model.

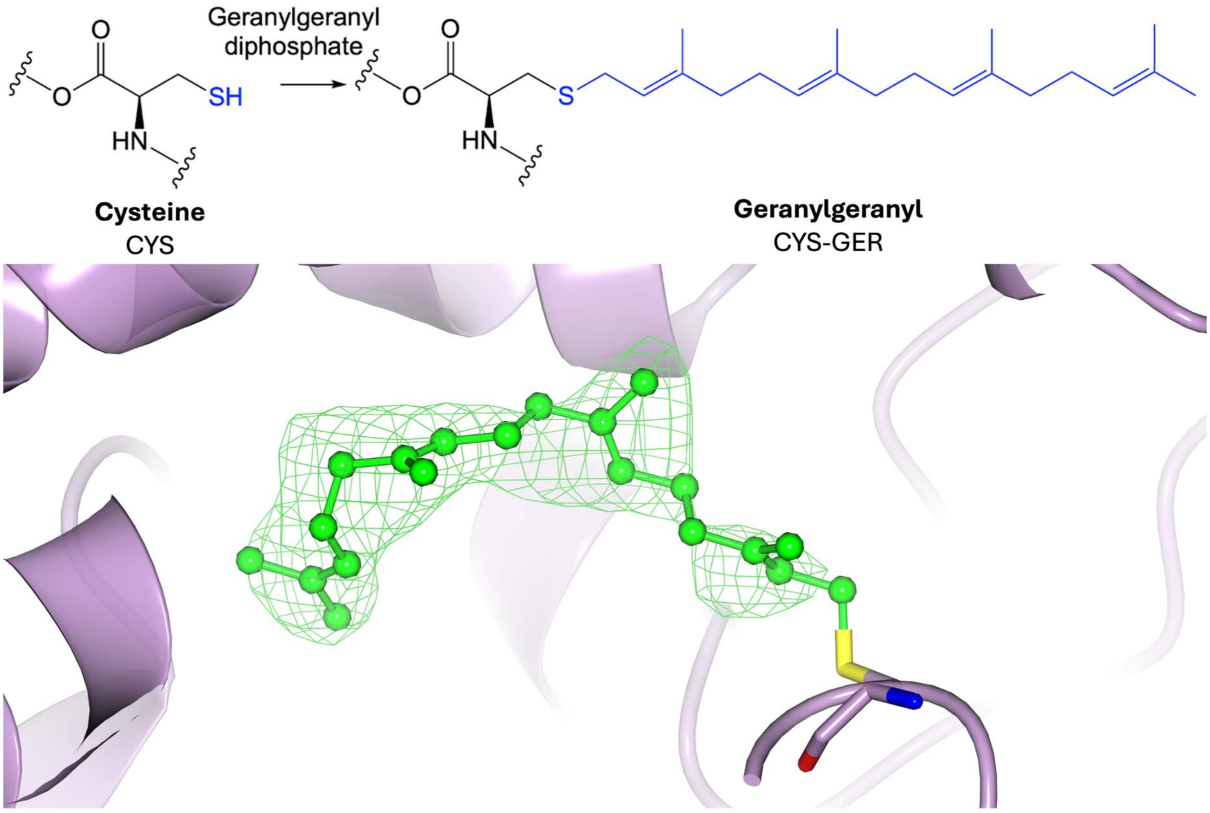

Figure S11 Prenylation. (Top panel) Prenylation of cysteine involves the addition of a geranylgeranyl group (or farnesyl group, Fig. 11) donated by geranylgeranyl diphosphate (or farnesyldiphosphate) to the side chain thiol group. (Bottom panel) Geranylgeranylcysteine (PDB code: 6J7F (S. Goto-Ito, A. Yamagata, Y. Sato & S. Fukai, unpublished work), CCD code: GER). Positive omit density is shown in green at 3σ for the modified residue. The rest of the protein chain is represented by a purple ribbon model.

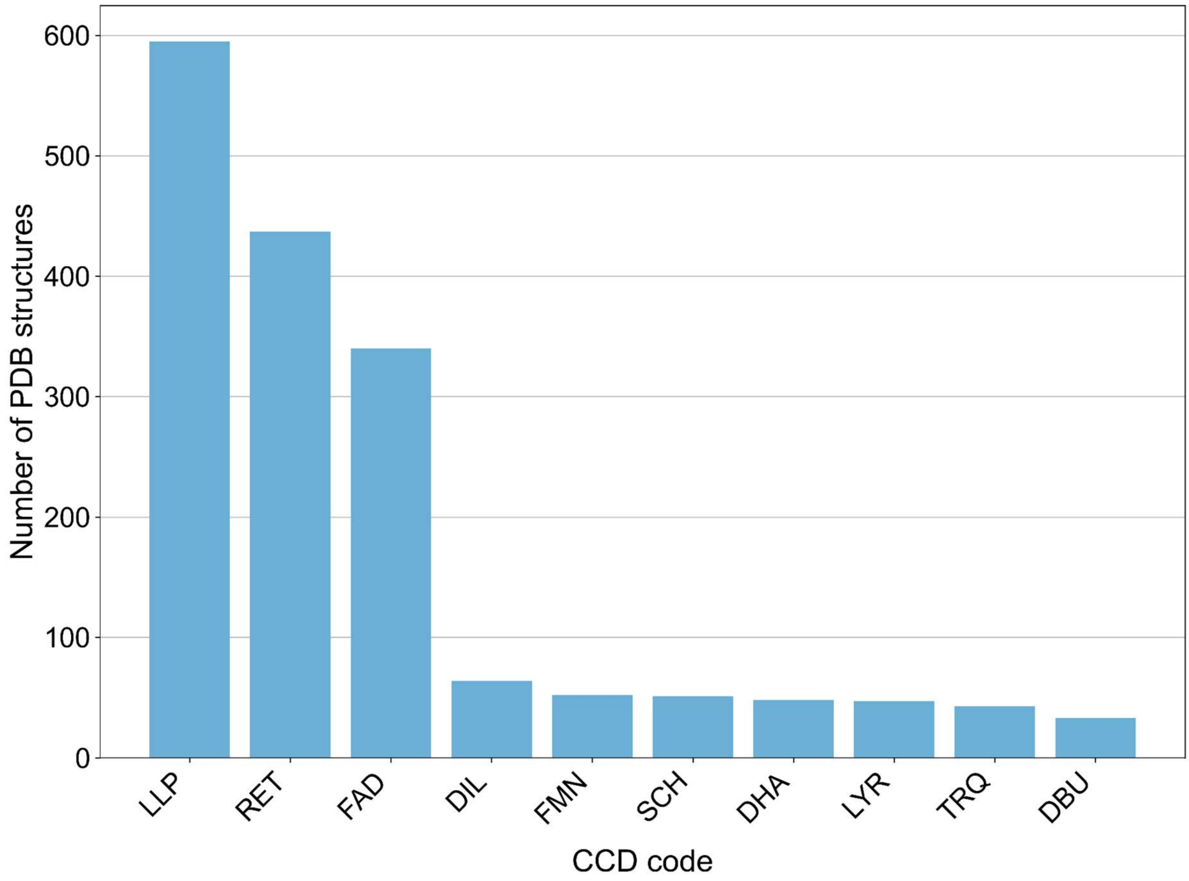

LLP = N6-(pyridoxal phosphate)lysine	RET = Retinal
FAD = Flavin-adenine dinucleotide	DIL = Chromophore (Met-Tyr-Gly)
FMN = Flavin mononucleotide	SCH = S-methyl-thio-cysteine
DHA = 2,3-didehydroalanine	LYR = N6-(retinylidene)lysine
TRQ = Tryptophylquinone	DBU = Dehydryobutyrine

Figure S12 Chemical modifications detected in the PDB. Dataset represents chemical modifications listed in the UniProt Knowledgebase PTM list that are not classified as PTMs. Data was obtained by searching the RCSB PDB Search API using identified CCD codes corresponding to PTMs. This data includes CCD codes which are located in the polymeric sequence and covalently linked to the polymer sequence.
